# Paraoxonase-1 (PON1) induces metastatic potential and apoptosis escape via its antioxidative function in lung cancer cells

**DOI:** 10.18632/oncotarget.17069

**Published:** 2017-04-12

**Authors:** Mark Borris D. Aldonza, Yeon Sung Son, Hye-Jin Sung, Jung Mo Ahn, Young-Jin Choi, Yong-In Kim, Sukki Cho, Je-Yoel Cho

**Affiliations:** ^1^ Department of Biochemistry, BK21 PLUS Program for Creative Veterinary Science Research and Research Institute for Veterinary Science, College of Veterinary Medicine, Seoul National University, Seoul, Republic of Korea; ^2^ Department of Thoracic and Cardiovascular Surgery, Seoul National University Bundang Hospital, Seoungnam-Si, Gyeonggi-Do, Republic of Korea; ^3^ Current address: Department of Chemical and Biomolecular Engineering, Korea Advanced Institute of Science and Technology (KAIST), Daejeon, Republic of Korea; ^4^ Current address: Bio Center, Incheon Technopark, Incheon, Republic of Korea; ^5^ Current address: College of Medicine, University of Ulsan, Seoul, Republic of Korea

**Keywords:** paraoxonase-1, PON1, lung cancer, cell death, HDL

## Abstract

Paraoxonase-1 (*PON1)* gene polymorphisms have been closely associated with the development of advanced cancers while PON1 secretion to the serum is linked with inhibition of oxidized high-density lipoprotein by its antioxidative function. Our group previously demonstrated that post-translational modification of serum PON1 in form of fucosylated PON1 is a potential biomarker of small cell lung cancer. Here, we interrogated the role of PON1 in the pathobiology of lung cancer (LC) by addressing cell-autonomous mechanisms using gain-of-function and loss-of-function approaches and protein expression profiling of tissue samples in our clinical biobank. PON1 expression in LC patient tissues varied between overexpression in squamous cell carcinoma and minimal loss in adenocarcinoma sub-types. Simultaneous overexpression of PON1 both at the gene and protein stability levels induced pro-oncogenic characteristics in LC cells and xenografts. PON1 overexpression supported metastatic progression of LC by decreasing G_1_/S ratio and LC cell senescence involving p21^Waf1/Cip1^. PON1 suppressed drug- and ligand-induced cell death and protected LC cells from genotoxic damages with maintained ATP levels, requiring p53-directed signals. PON1 promoted ROS deregulation protecting the mitochondria from dysregulation. PON1 knockdown resulted in the blockage of its antioxidant function in LC cells through Akt signaling with reduced invasive signature as a consequence of scant expression. Targeted glycolysis stimulated PON1 antioxidant activity regulating phosphorylation of AMPK-α. The functional data imply that exploitation of the antioxidative function of PON1 is consequential in driving LC pathogenesis at the cell-autonomous mechanistic level with consequences on tumor growth.

## INTRODUCTION

At the initial stage of carcinogenesis, cancer cells highly regulate the uptake of circulating lipoproteins mediated by binding receptors to maintain cell metabolism and homeostasis [1, 2]. Redox signaling plays a vital role in malignant tumor cell transformation through reactive oxygen species (ROS)-mediated function. Dysregulation of this specific redox utility results in the development of various diseases including cancers [3]. Importantly, dysfunctions in the expression of high density lipoprotein (HDL)-bound proteins affect antioxidative functions in various cancers with its macrophage cholesterol efflux function as a critical player [4]. Clinical studies have shown that low serum HDL cholesterol, which serves as a carrier-delivery platform for lipoprotein-bound proteins to the circulation, is associated with acquired advanced lung cancer (LC) incidence [5, 6].

Paraoxonase-1 (PON1) is an HDL-binding protein with antioxidant properties, along with two other paraoxonases PON2 and PON3 [7]. PON1 is bound to circulating HDL particles and is known to enzymatically hydrolyze organophosphates involved in lipid metabolism of diseases [8]. The function of PON1 is anticipated in cancer disease models since it is found that certain PON1 genotypes were prone to be a cancer risk factor such as the occurrence of single nucleotide polymorphisms (SNPs) in variant alleles of PON1 [9, 10]. In cancer, PON2 and PON3′s association is based on their up-regulated expressions in various tumors with anti-apoptotic and protective effects in the mitochondria from several chemical-mediated dysfunctions [11]. Nevertheless, all PONs are implicated in the pathogenesis of several inflammatory diseases including atherosclerosis, diabetes, and cancer. Since it is reported that the pro-inflammatory cytokines such as IL1β and TNF downregulate PON1 expression and secretion to serum by liver cells, it is likely that the lower levels of PON1 in serum can be a result of unhealed long-lasting inflammatory conditions in LC patients. Collectively, addressing PON1 needs to be completed so we understand better the roles of PONs in redox system and cell death regulation in cancers.

Our understanding of the molecular basis of PON1-implicated cancer pathogenesis has been limited by the scarcity of mechanistic studies that look beyond PON1 genetic polymorphisms and enzyme activity. To address this lack thereof, we seek to dissect, at the cell-autonomous level, whether PON1 displays oncogenic characteristics primed by its antioxidative function. We examined PON1 protein expression in LC tissues of human patients and corroborated this with *PON1* copy number analysis using TCGA datasets of human LC tumors. Then we further elaborated the consequence of PON1 regulation in LC cells and tumor xenografts and that the exploitation of its antioxidative function can impact tumorigenesis and escape from cell death. Our study reveals that overexpression of PON1 intracellularly can stimulate LC cell outgrowth and induce anti-apoptotic effects through antioxidative function regulating ROS and glycolytic metabolism while PON1 suppression can reduce Akt-directed cell metastasis. We present evidence for PON1 having anti-apoptotic and anti-oxidative features in LC cells eliciting tumor growth.

## RESULTS

### Varied PON1 protein and gene expressions in lung cancer tumor tissue sub-types and lung cancer cell lines

**Figure 1:**
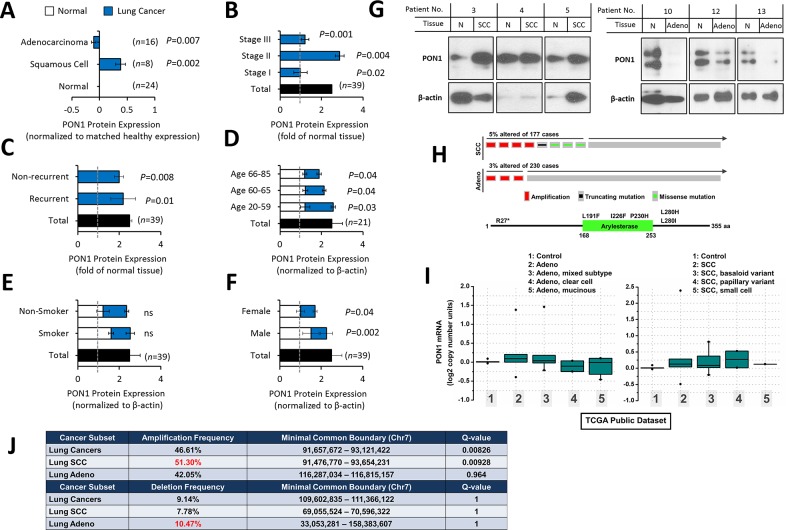

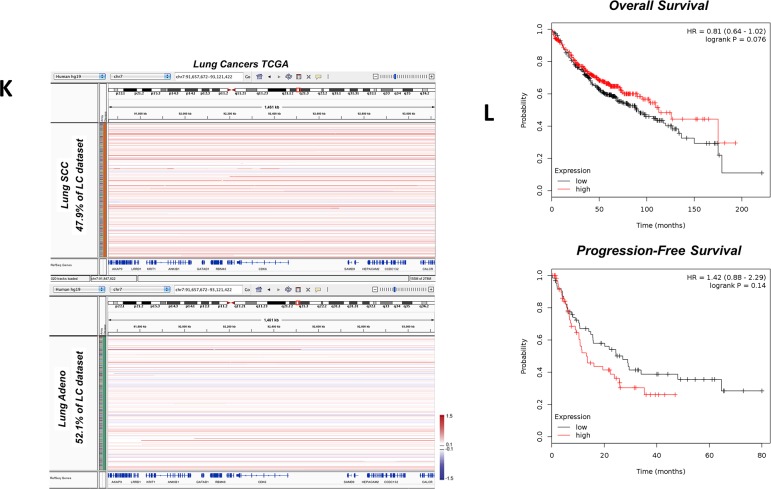
Variation of PON1 protein and gene expression between lung squamous cell carcinoma and lung adenocarcinoma patients **(A)** Densitometry analysis of lung cancer subtype tissue-based expression of PON1 protein from matched clinical pairs of lung squamous cell carcinoma and lung adenocarcinoma normalized to adjacent normal control. **(B, C, D, E, F)** Matched tissue samples were either summarized (total) or separated into different groups of: stage, recurrence, age, smoking history, and gender. Presented results were summarized from two biological replicates of the blotting experiment. **(G)** Representative blots of PON1 protein patient tissue expression selected from 39 matched cases. β-actin variations apparent in some blots were considered in calculating relative densitometry values for each blot using nonparametric Kruskal-Wallis statistical test. **(H)** Genetic alterations and mRNA expression changes (copy number) of *PON1* in the TCGA dataset of lung cancer samples. *PON1* genes are represented in rows, and individual cases or patients are represented as columns (upper panel). Higher magnification images are shown in the inset (lower panel). These oncoprints are based on data obtained from the cBio portal (http://www.cbioportal.org). **(I)** TCGA cohorts of lung cancer (lung squamous cell carcinoma and lung adenocarcinoma variants) gene expression study were analyzed using Oncomine (http://www.oncomine.org). **(J)** Characterization of amplification and deletion of the *PON1* locus. Summary of both amplification and deletion frequency and boundary of alteration of the *PON1* locus in the lung squamous cell carcinoma and lung adenocarcinoma tumor types. Data were obtained through query of the TCGA TumorScape database (Broad Institute). **(K)** Lung squamous cell carcinoma and lung adenocarcinoma patients exhibiting amplification at the *PON1* locus were visualized using the Integrative Genomic Viewer (IGVv2.3; Broad Institute). Red bars represent amplification, blue bars represent the degree of deletion, and white bars represent no alteration. DNA copy number ratio is relative to a reference somatic DNA sample. It must be noted that the single patient representing a focal amplification/deletion at the *PON1* locus (boxed in red in the profile graphs). **(L)** Correlation of *PON1* expression with patient survival in lung adenocarcinoma. *PON1* expression was stratified as high versus low against median expression. Graphs were plotted using kmPlotter (www.kmplot.com) with overall survival and progression-free survival within previously published datasets. Tissue protein samples from our cohorts were blotted three times and were biologically repeated three times

Tissue-based protein expression analysis revealed that PON1 has a varied expression pattern between squamous cell carcinoma (SCC) and lung adenocarcinoma tissues. In 8 matched cases, SCC tissues revealed a shallow overexpression (in densitometry) than adjacent normal tissues, while in 16 matched cases of adenocarcinoma, PON1 is minimally decreased (Figure 1A). Clinico-pathological information-based categorization of the 39 matched tissues (Table 1) affirmed higher PON1 protein expression at LC stage II than in stages I and III (Figure 1B). LC tissues of recurrent and non-recurrent groups showed no significant difference (Figure 1C), but this can be a result of our limited sample cohorts of SCC (non-recurrent: 7 cases; recurrent: 3 cases) and lung adenocarcinoma (non-recurrent: 16 cases; recurrent: 13 cases). PON1 is slightly up-regulated in younger age-group of 20-59 compared to older groups of 60-65 and 66-85 both in LC tissues (Figure 1D). Slight differences were observed between normal and LC tissues of patients with or without smoking history (smoker) and non-smokers (Figure 1E), and of between female and male patients (Figure 1F), respectively. Representative blots of PON1 protein expression in LC tissues are shown in Figure 1G. To corroborate the varied PON1 expression between SCC and adenocarcinoma, we analyzed a larger dataset obtained from cBioPortal for Cancer Genomics (http://cbioportal.org). A separate TCGA provisional cohorts of lung SCC and adenocarcinoma samples show higher amplification of DNA copy numbers in SCC with truncating and missense (putative passenger) mutations (Figure 1H). PON1 gene expression profiles were further examined in public datasets from Oncomine database (http://www.oncomine.org/) where we utilized a TCGA lung cancer cohort showing normal versus cancer copy number analysis. In the adenocarcinoma cohort, PON1 is slightly amplified in general lung adenocarcinoma samples (261 samples) and mixed subtype lung adenocarcinoma (67 samples) but deleted in lung clear cell adenocarcinoma (2 samples) and lung mucinous adenocarcinoma (6 samples) (Figure 1I, left panel). In the lung SCC cohort, PON1 has relatively high amplification of DNA copy numbers in all SCC variants (348 general SCC samples; 8 SCC, basaloid variant samples; 2 SCC, papillary variant samples; 1 SCC small cell variant sample) compared to both lung normal (no value) and the adenocarcinoma cohort (Figure 1I, right panel). Similar patterns were observed using other available LC cohort datasets showing higher amplified copy numbers in SCC than in adenocarcinoma (Supplementary Figure 1A, 1B, 1C). We wondered whether this expression pattern in human tumors would persist in larger TCGA datasets. We examined copy number variations for human PON1, which lies in a broad region on chromosome 7q21.3 where a cluster of three related paraoxonase genes are located. GISTIC analysis reveals that PON1 has infrequent amplification and deletion across the entire TumorScape/TCGA dataset of 9,000+ tumors (http://www.broadinstitute.org/tcga/). Although infrequent, we were able to observe higher amplification frequency in SCC than adenocarcinoma and higher deletion frequency in adenocarcinoma than SCC (Figure 1J). Supporting this, we were able to observe higher focal, specific amplification at the *PON1* locus in SCC than in adenocarcinoma (Figure 1K). These show high PON1 variability patterns across different LC types. We next examined how *PON1* can possibly impact patient outcome in annotated datasets of human NSCLC tumors. Utilizing a published meta-analysis of LC microarray datasets that assessed prognostic values of biomarkers using transcriptomic data [12], we found that low *PON1* expression in lung adenocarcinoma patients is correlated with decreased overall survival while associated with improved progression-free survival (Figure 1L). This suggests that varied *PON1* mRNA expressions across LC subtypes may correlate with increased or decreased metastatic frequency as survival data from the mined cohorts is largely due to metastasis.

**Table 1:** Clinical and pathological patient characteristics of the human tissue samples used in this study

Sample No.	Age	Sex	Smoking History	Cell Type	TNM	Stage	Recurrence	Recurrence-free period (month)
1	22	M	C (5)	Adeno	U.K.	2	-	26
2	52	F	N	Adeno	1/0/0	1	-	22
3	80	M	C (60)	SCC	2/0/0	1	-	26
4	71	M	C (120)	SCC	3/0/0	1	-	26
5	71	M	C (100)	SCC	2/0/0	2	-	22
6	70	M	C (30)	SCC	3/1/0	2	-	26
7	55	M	F (1.5/2)	Adeno	3/0/0	1	-	22
8	57	M	C (60)	SCC	1/0/0	1	-	24
9	60	M	N	Adeno	U.K.	1	-	25
10	76	F	N	Adeno	U.K.	1	-	25
11	56	M	F	Adeno	2/0/0	1	+	13
12	75	M	N	Adeno	5/2/0	3	+	8
13	45	F	N	Adeno	3/1/0	2	+	13
14	63	F	N	Adeno	U.K.	1	-	26
15	75	M	F (25/25)	Adeno	3/0/0	1	-	25
16	72	M	F (10/10)	Adeno	1/0/0	1	+	19
17	77	M	F (16/30)	Adeno	1/0/0	1	-	23
18	65	M	F (12/30)	SCC	3/0/0	1	-	23
19	67	M	N	Adeno	3/0/0	1	-	23
20	68	F	N	Adeno	3/0/0	1	-	22
21	72	M	F (10/10)	Adeno	1/0/0	1	+	19
22	66	M	N	SCC	3/0/0	1	+	7
23	67	F	N	Adeno	3/0/0	1	+	15
24	64	M	C (23)	SCC	2/0/0	1	+	13
25	76	M	F (6/30)	SCC	4/0/0	2	+	18
26	43	F	N	Adeno	3/2/1	3	+	13
27	45	M	C (20)	Adeno	3/0/0	3	+	7
28	82	M	N	Adeno	2/1/0	2	+	13
29	56	F	N	Adeno	3/2/0	3	+	6
30	78	F	N	Adeno	5/0/0	2	-	20
31	62	M	C (45)	Adeno	4/0/0	2	+	19
32	70	F	N	Adeno	2/0/0	1	-	22
33	67	M	F (20/25)	Adeno	3/0/0	1	-	22
34	60	M	F (8/30)	Adeno	1/0/0	1	-	22
35	71	F	F (3/3)	Adeno	1/0/0	1	+	18
36	65	M	F (8/20)	Adeno	1/0/0	1	+	14
37	70	F	F (3/1.5)	Adeno	4/0/0	1	-	22
38	84	M	F (14/45)	SCC	3/0/0	1	-	23
39	69	M	F (2.5/50)	Adeno	U.K.	2	-	23

Abbreviations used: Adeno; adenocarcinoma, SCC; squamous cell carcinoma, N; never smoked, F; former smoker, C; current smoker, U.K.; unknown, -; no recurrence at present, +; recurrence at present.

**Figure 2:**
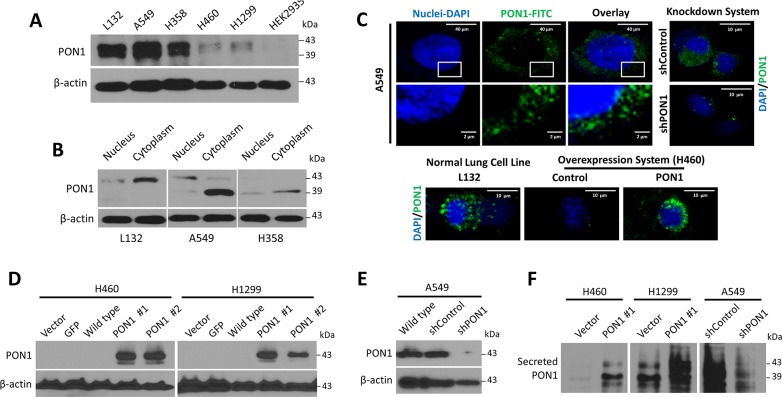
PON1 is located in the cytosolic fraction and is differentially expressed in various lung normal and tumor cell lines **(A)** PON1 protein expression in different lung normal (L132) or cancer cell lines (A549, H460, H1299, H358) including another normal cell line (HEK-293). **(B)** Western blot analysis of nuclear and cytosolic cell extracts (50 μg of each fraction). **(C)** Live A549 and A549-shPON1 cells stained with reduced FITC (pseudocolored in green) and DAPI. Cells were analyzed by confocal microscopy. Scale bars = 40 μm or 2 μm as indicated. **(D, E)** Stable overexpression and knockdown of PON1 by lentiviral-induced systems were examined by Western blotting. In PON1 overexpression system (H460- and H1299-PON1), two isoforms of stable PON1 overexpression (#1 and #2, respectively) differ in lentiviral particles used for transfection. **(F)** Immunoprecipitation of cells with anti-human PON1 antibody. Representative examples of three independent experiments are shown. All experiments were conducted at least in triplicate and biologically repeated at least twice. Statistical analysis was performed using unpaired Student's t test.

In cells, PON1 expression varied with high expression in lung epithelial cell line L132 and in some LC cell lines A549 and H358, whereas PON1 expression was barely detected in other LC cell lines, H460 and H1299, which appeared, if any, as higher molecular weight form of 43 kDa (Figure 2A). PON1 appeared as double-band at 39 and 43 kDa with higher expression in the cytosol and little or no expression in the nucleus of L132, A549 and H358 cells, with major 39 kDa band in A549 and H358, whereas a 43 kDa band appeared in L132 cells, both dominantly in cytoplasm (Figure 2B). It is not reported thus far the functional differences of 43 kDa and 39 kDa PON1 proteins, although it is postulated that 43 kDa is a glycosylated form and 39 kDa is not [13]. Protein function depends on localization. Although it is suggested that extracellular PON1 can be taken up to cell cytoplasm [14] PON1 distribution was localized in the overlapping borders of nuclear membrane in live A549 cells with likewise condensed, granular pattern in nucleus and cytosol of H460 cells when forced to overexpress PON1 while more widely scattered in the cytoplasm of L132 cells (Figure 2C). PON1 was significantly overexpressed by bicistronic lentiviral vector-mediated stable transduction in H460 and H1299 cells as higher 43 kDa forms (Figure 2D; Supplementary Figure 2A). A549 cells infected with lentivirus-encoding PON1 shRNA (A549-shPON1) appeared to have efficient PON1 knockdown (compared to scrambled shRNA control; shControl) with almost no protein expression, and with >2-fold decrease in mRNA expression (Figure 2E; Supplementary Figure 2B). PON1-overexpressing H460-PON1 and H1299-PON1 cells have high PON1 secretion while A549-shPON1 cells have very low secretion, all displaying 39 kDa and 43 kDa forms (Figure 2F). We observed both 39 kDa and 43 kDa forms of PON1 in the secretion of A549, H460-PON1, and H1299-PON1 cells.

### PON1 drives lung cancer cell growth, regulates cell cycle progression, and supports senescence blockade

**Figure 3:**
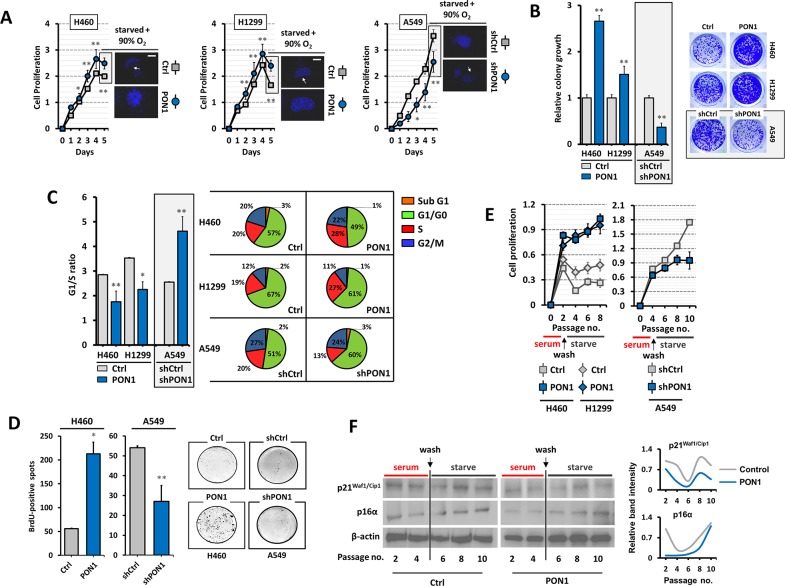
PON1 regulation impacts lung cancer cell growth and arrest programs **(A)** Assessment of cell viability. Before day 5, cells were serum starved for 24 h and were subjected to DAPI staining. Subsequent to serum starvation, cells were pre-exposed to 90% O_2_ level for 12 h and incubated in normoxia for 12 h (displayed as immunofluorescence in right panel of each graph). **(B)** Colony formation (>50 cells) was assessed by crystal violet staining. **(C)** Mean G_1_/S-phase ratio from cell cycle analysis (left panel) and cell cycle progression fractions of >15,000 cells analyzed by FACS (right panel). **(D)** BrdU-positive spots of cells examined at day 4 at early cell passage in serum-starved medium. **(E)** Cell passage-dependent viability after pre-culture of cells at low density for 3 days in serum-rich media containing low etoposide concentration and then subsequently starved them for another 6 days. SA-β-gal assay was used to confirm presence of senescent cells after recovery (data not shown). **(F)** Western blot analysis of whole cell lysates of same samples from E. β-actin served as the loading control. *P<0.05. Error bars are mean ± S.E.M. n=3~5. All experiments were conducted at least in triplicate and biologically repeated at least twice. Statistical analysis was performed using unpaired Student's t test.

Although it is suspected that antioxidative function of PON1 contribute to cancer development, it currently lacks detailed mechanism to prove such. Knockdown of PON1 in A549-shPON1 cells displayed robust decrease in proliferation rate overtime while markedly increased when forcedly expressed in H460-PON1 and H1299-PON1cells with less nuclear condensation accompanied with enlarged nuclei size under serum-starvation and pre-exposure to 90% oxygen (Figure 3A; Supplementary Figure 2C). Colony formation was dramatically increased in H460-PON1 and H1299-PON1 cells while reduced in A549-shPON1 cells (Figure 3B). We next assessed the probable influence of PON1 regulation in cell cycle progression using FACS. Both PON1-overexpressing cells were ≥27% in S phase and >49% in G_1_/G_0_ phase compared with their controls (19-20% in S phase and 57-67% in G_1_/G_0_), and thus decreasing the G_1_/S ratio approximately >1.3 fold explaining the rapid proliferation, in contrast with 8% lesser S phase and >9% greater G_1_/G_0_ fractions when PON1 was knocked down causing cells to be arrested within 3 days (Figure 3C). Supporting this claim, PON1-overexpressing cells incorporated more BrdU while obstructed when PON1 was suppressed (Figure 3D; Supplementary Figure 4A, 4B). This reinforces a causal possibility of PON1-inflicted cellular senescence blockade. Thus, we tested whether PON1 supports escape from senescence-defining permanent growth arrest upon induction. To undermine this process, we cultured the cells at low cell density in serum-rich media and treated them with low etoposide concentration (3 μM) at day 3, washed the cells at day 5, starved them for another 4 to 6 days (serum-starvation; pre-exposed to 90% oxygen) until we observed proliferation arrest for at least 3 subsequent passages and confirmed a cellular senescence phenotype by SA-β-gal activity (data not shown). PON1-overexpressing cells maintained the growth pattern while control cells dramatically decreased in proliferation, in contrast with A549-shPON1 cells, which reached later passages of 8 to 10 with diminishing proliferation rate (Figure 3E). Ensuring this senescence blockade, PON1-overexpressing H460 cells displayed down-regulated p21^Waf1/Cip1^, which triggers growth arrest associated with senescence (Figure 3F; normalized band intensity values in right panel), while not significantly affecting p16α expression. Interestingly, under normal culture, transient PON1 overexpression (by pCMV-GFP-PON1 transfection) up-regulated p16 and cyclin E gene levels among others (Supplementary Figure 4A, 4B, 4C).

### Antioxidative function of PON1 is maintained in ROS-dependent manner

**Figure 4:**
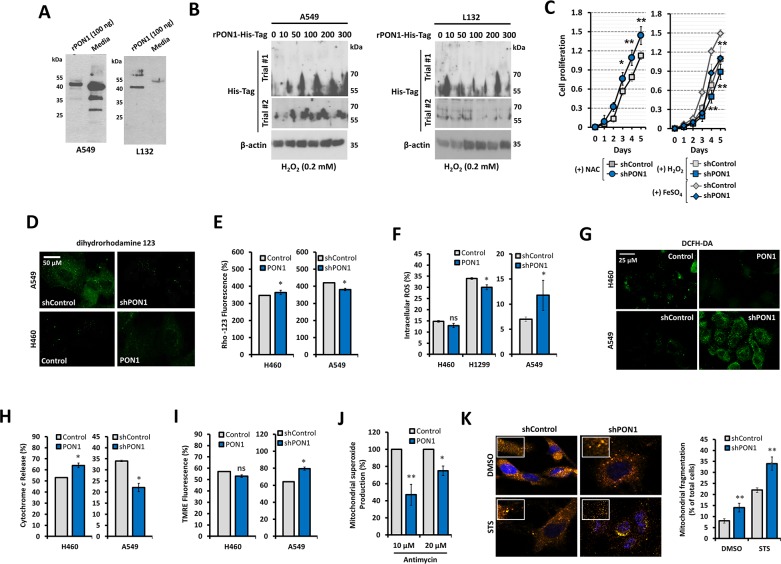
PON1 regulation positively impacts lung cancer by antioxidative function-controlled ROS accumulation **(A)** PON1 immunoblot of A549 (left panel) and L132 (right panel) cells after loading with 100 ng of human rPON1 protein for 24 h. **(B)** Detection of human rPON1-His-tag protein using His-tag antibody in A549 cells (left panel) and L132 cells (right panel) under oxidative stress induced by H_2_O_2_ for 24 h. **(C)** Assessment of cell viability after treatment with NAC, H_2_O_2_, or FeSO_4_. **(D)** Representative images showing fluorescence signal after loading with dihydrorhodamine 123. Scale bars = 50 μm. **(E, F, G, H, I)** Cells were analyzed by FACS for the following: ROS determination (both rhodamine and DCFH fluorescence intensity units), dihydrorhodamine oxidation fluorescence, cyctochrome *c* release, and mitochondrial membrane. FACS-analyzed cells were 400-1000 in population. In G, representative images showing fluorescence signal after loading with DCFH-DA. Scale bars = 25 μm. **(J)** FACS analysis of cells loaded with Mito-HE (2 μM), treated with antimycin A (10 and 20 μM). **(K)** Mitchondrial fragmentation determined by MitoSOX (pseudocolored in orange; 10 μM) after loading cells with either 0.01% DMSO or 1 μM STS and quantification of fragmented cells. Bar graphs mean ± S.E.M. of total fragmented cells. All experiments were conducted at least in triplicate and biologically repeated at least twice. Statistical analysis was performed using unpaired Student's t test and ANOVA. Symbols represent mean ± S.E.M. n = 3~7; n.s., not significant; *P<0.05; **P<0.01.

PON1 works as an antioxidant enzyme with HDL-associated macrophage differentiation regulatory activity [14]. However, it is not well documented whether its anti-oxidative function is maintained in cancers. To define the antioxidative regulatory activity of PON1 in LC, we first determined whether PON1 expression can be regulated by oxidative stress and defined the regulatory involvement of ROS in PON1-driven cell growth. PON1 internalization from the extracellular matrix (ECM) to cellular compartment was potentially detected when recombinant PON1-tagged with His (rPON1-His) was admixed in the media. Despite the wild-type PON1 expression in both cells, we observed higher uptake of rPON1-His in A549 cells than L132 cells (Figure 4A). Interestingly, this uptake persisted upon H_2_O_2_-induced oxidative stress in A549 cells but not in L132 cells supporting that PON1 uptake can be an adaptive response mechanism of cancer cells to oxidative stress (Figure 4B). To directly examine the consequence of rPON1-His uptake from the media on cells, we allowed cells to be fed with rPON1-enriched media for 24 h and subsequently induced oxidative stress with increasing concentrations of H_2_O_2_. Notably, there was a higher rescue frequency of viability in A549 than L132 cells (Supplementary Figure 5). Interested in ROS-directed promotion of Akt-regulated response to oxidative stress, we detected a recovery of ablated growth rate of A549-shPON1 cells after treatment with an antioxidant N-acetyl-cysteine (NAC) while further inhibited when exposed to ROS-stimulators, H_2_O_2_ and FeSO_4_ (Figure 4C). Reflecting this functional input on mitochondrial dysfunction, we observed that after loading with dihydrorhodamine 123 (DHR-123), the fluorescence output was suppressed in A549-shPON1 cells while increased in H460-PON1 cells (Figure 4D), suggesting PON1 exploits the energization of oxidative stress supplied by mitochondria where DHR-123 is concentrated upon conversion to rhodamine 123 (RHO-123), which appeared to have lower mean fluorescent intensity (MIF) in response to PON1 loss (Figure 4E), negatively regulating mitochondrial ROS production but independent of ROS scavenging process because upon inhibiting ROS scavenging activity by CDNB (1-chloro-2,4-dinitrobenzene), the same results occurred (Supplementary Figure 6). Knockdown of PON1 increased the MIF of dichloro-dihydro-fluorescein diacetate (DCFH-DA) while lowered when forcedly overexpressed (Figure 4F, 4G). These findings argue that while downregulation of PON1 enhances ROS production reflecting PON1 to have antioxidant activity, forced overexpression of PON1 can behave as both prooxidant and antioxidant which might be dependent on mitochondrial energization by removing superoxide anion or generating hydrogen peroxide. Confirming this hypothesis, PON1 knockdown caused a decrease in the level of cytochrome *c* release from the mitochondria to cytosol with elevated mitochondrial membrane potential measured by TMRE (tetramethylrhodamine ethyl ester), while overexpression caused an increase (Figure 4H, 4I). Under brief treatment with Antimycin A (AA), superoxide levels were diminished in PON1-overexpressing cells (Figure 4J). PON1 knockdown increased the staurosporine (STS)-mediated induction of mitochondrial fragmentation, indicating an impeded mitochondrial fusion affecting GTPase-related proteins at mitochondrial membranes (Figure 4K). Thus, all together these data suggest that PON1 antioxidant activity can be maintained through peroxidase-type mechanism in mitochondria regulating ROS in lung cancer cells.

### PON1 deprivation causes drawback of metastatic behavior in lung cancer cells through antioxidative response

**Figure 5:**
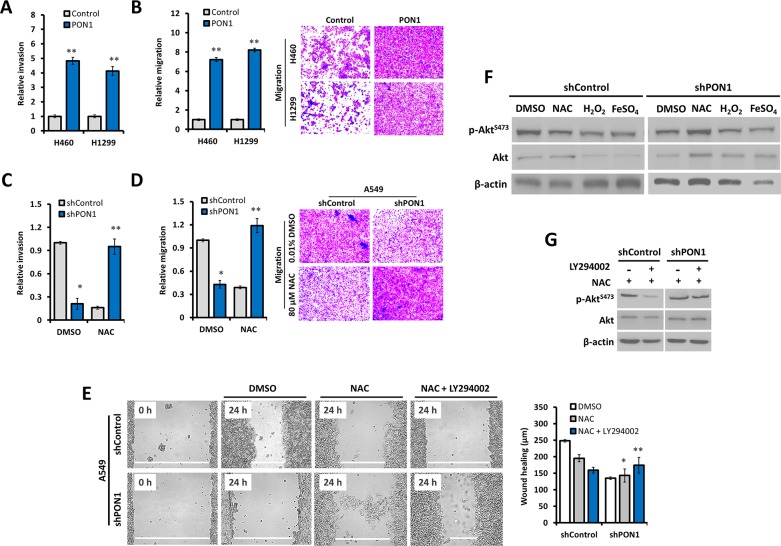
PON1 controls metastatic lung cancer cell behavior by regulating oxidative response **(A, B)** Invasion and migration counts per field of PON1-overexpressing H460-PON1 and H1299-PON1 cells versus vector control cells for 24 h. **(C, D)** Invasion and migration counts per field of PON1-knocked down A549-shPON1 cells versus shControl cells for 24 h. **(E)** Scratch-wound healing ability of cells after pre-exposure to 0.01% DMSO, 10 mM NAC, or 10 mM NAC in combination with 10 μM Akt-inhibitor LY294002. Representative bar graphs show the width in μm of scratch recorded. Representative migration micrographs are shown taken using phase-contrast microscope. **(F, G)** Western blot analysis of whole cell lysates. Cells were treated with 0.01% DMSO, 10 mM ROS-inhibitor NAC, 1 mM ROS-stimulators H_2_O_2_ and 7 mM FeSO_4_. Cells were treated with or without combination of NAC and Akt-inhibitor LY294002 for G. All experiments were conducted at least in triplicate and biologically repeated at least twice. Statistical analysis was performed using unpaired Student's t test.

To examine how our observed PON1-controlled antioxidative response in LC cells impacts cell behavior, we examined cell migration and invasion. We hypothesized that the regulated ROS directed by PON1 could affect hostile mobility of LC cells since ROS can govern LC cell motility with delimited mitochondrial defects associated with Akt [15, 16]. Wild-type NSCLC cell lines showed different ROS status and aggressiveness (Supplementary Figure 7). We then used this finding as a baseline to our phenotypic conclusions in the cell lines we tested. PON1 induced rapid chemotaxis-directed migration rate in LC cells with increased invasiveness (Figure 5A, 5B). NAC-induced ROS depletion significantly increased migration and invasion rates of PON1-deficient cells (Figure 5C, 5D), while ROS stimulation or suppression regulated the aggressive growth of stably PON1-overexpressing cells (Supplementary Figure 8). Akt inhibition by LY294002 in NAC-induced ROS depletion markedly increased migration rate of live PON1-knockdown cells (Figure 5E), with PON1 ablation increasing Akt phosphorylation when ROS is scavenged or decreased when stimulated. Recovery of Akt phosphorylation in PON1-knockdown cells was achieved when Akt and ROS were both inhibited (Figure 5F, 5G). Given the role of Akt in the up-regulation of the *PON1* gene expression, this suggests that PON1 positively impacts Akt-implicated metastatic capacity [17].

### Targeted glycolysis stimulates PON1-dependent antioxidative response in lung cancer cells

**Figure 6:**
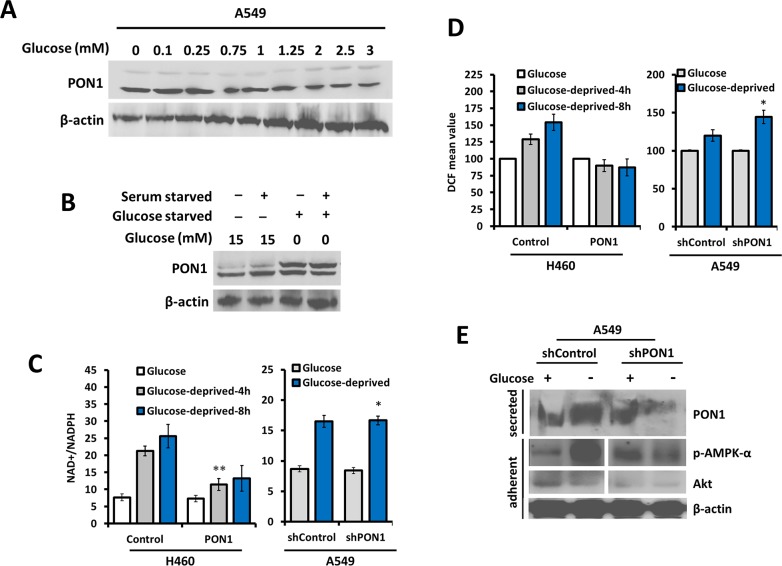
Glycolytic regulation controls PON1-dependent antioxidative response in lung cancer cells **(A)** Immunoblot detection of PON1 from whole cell lysates after glucose-enrichment in the media with indicated concentrations for 24 h. **(B)** Cells were serum-starved, glucose-starved, or glucose-enriched prior to Western blotting with PON1 antibody. **(C)** Homogenous detection of oxidized and reduced NAD+ and NADPH in H460-PON1, H1299-PON1, A549-shPON1, and their respective controls as indicated after the transfected cells have been exposed to the indicated glucose-deprivation periods. **(D)** ROS production after cells were either enriched with glucose or glucose-deprived. **(E)** Immunoblotting and immunoprecipitation of cells after glucose-enrichment or glucose-deprivation. Three independent experiments were summarized. Symbols represent mean ± S.E.M. n = 3~7; n.s., not significant; *P<0.05; **P<0.01.

PON1-deficient mice are found to have decreased glycolysis [18]. Glucose-enrichment invariably regulated PON1 expression while glucose-starvation recovered the expression in A549 cells (Figure 6A, 6B) while no regulation was observed in L132 cells (data not shown). PON1 overexpression negatively regulated NAD+/NADPH levels with significant decrease in production upon glucose-deprivation compared to control wild-type cells while scant PON1 in A549-shPON1 cells positively regulated NAD+/NADPH levels with significant increase upon glucose-deprivation compared to control cells expressing wild-type PON1 (Figure 6C). Moreover, PON1 overexpression suppressed glucose-deprivation-induced ROS production, while shPON1 reversed this occurrence (Figure 6D). To further explain what is driving this mechanism, we examined the possible role of AMPK. AMPK mediates glycolysis in cancer metabolism [19]. Glucose-starvation induced the phosphorylation of AMPK-α in A549-shControl cells while less expressed in A549-shPON1 cells with scant Akt expression. Along with this, secretion of PON1 appeared to be mediated by a glucose-directed mechanism (Figure 6E). Thus, it is deducted that glucose deprivation induces ROS that activates AMPK by phosphorylation, which then increases PON1. The elevated PON1 thus suppresses ROS in lung cancer. These all together suggest that secretion of PON1 might involve AMPK to direct the glycolysis-mediated action affecting PON1 release and influence PON1-directed antioxidative function.

### PON1 supports escape from therapy-induced apoptosis in lung cancer cells

**Figure 7:**
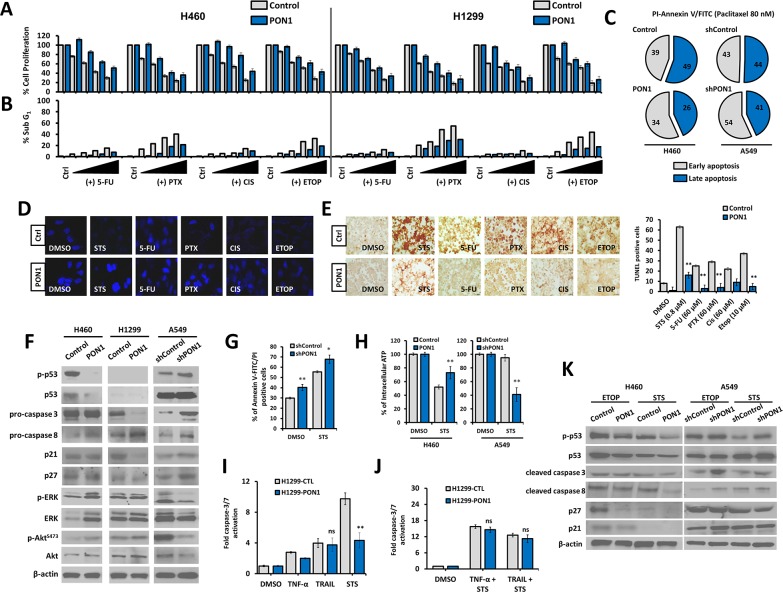
PON1 supports escape from drug- and ligand-induced cell death in lung cancer cells **(A)** Cell growth inhibition rate after binary dose-dependent treatment with chemotherapeutics. **(B, C)** FACS-based detection of drug-induced apoptotic fraction (sub-G_1_ phase) of cells and identification of early and late apoptosis. Symbols represent mean ± S.E.M. **(D)** Upon induction of apoptosis by various indicated chemotherapeutic drug (0.8 μM STS, 60 μM 5-FU, 60 μM PTX, 60 μM CIS, 10 μM ETOP) for 16 h, cells were assessed for nuclear morphology by DAPI staining shown as representative fluorescent images. **(E)** The same cells and drug treatment procedures were used in TUNEL assay as in D. Bar graph (right panel) shows representative total count of brightly stained nuclei of apoptotic cells. **(F)** Western blotting of cells to examine endogenous levels of total and phospho-p53, pro-caspases 3 and 8, p21, p27, total and phospho-ERK, and total and phospho-Akt. β-actin served as the loading control. **(G)** Cells were either treated with 0.01% DMSO or 1 μM STS and analyzed for Annexin-V-FITC/PI by FACS. **(H)** Cells were treated with either 0.01% DMSO or 5 μM STS for 48 h and assessed for intracellular ATP levels. **(I, J)** Cells were treated with 200 U TNF-α, 30 ng/μL TRAIL, 1 μM STS for 16 h, individually or both in combination with STS, and assayed for caspase-3/7 activation. **(K)** Cells were loaded with either 20 μM ETOP or 2 μM STS for 8 h and subjected to protein blotting with antibodies against total and phospho-p53, cleaved caspases 3 and 8, p27, and p21. β-Actin served as the loading control. Images and figures were selected as representative data from three independent experiments. Three independent experiments were summarized. Symbols represent mean ± S.E.M. n = 3~7; n.s., not significant; *P<0.05; **P>0.01.

We next tested the impact of PON1 overexpression or knockdown on therapeutically induced LC cell death using STS, 5-fluorouracil (5FU), paclitaxel (PTX), cisplatin (CIS), and etoposide (ETOP). PON1 afforded protection from drug-induced cell viability inhibition (Figure 7A; H460 data in right panel and H1299 data in left panel) and lesser sub-G_1_ population (Figure 7B). In addition, regulation of PON1 inflicted a difference in the ratio of early (EA) and late apoptotic (LA) fractions induced by PTX in LC cells with a decrease in EA with subsequent significant decrease in LA fraction in PON1-overexpressing cells (overall reduction in apoptotic cells) while increase in EA in PON1 knocked down cells (Figure 7C), supporting the data showing that loss of PON1 increased STS-mediated ligand-induced cell death with increased LA fraction (Supplementary Figure 9). Consistent with this, maintained nuclear morphology was observed compared to control with either condensed and/or fragmented nuclei after pre-exposure to drugs (Figure 7D) with relatively less TUNEL-positive cells revealed by incorporation to nuclei of dying LC cells (Figure 7E). Corresponding to these events we turned to the cell death pathway to examine probable mechanistic consequences. In LC, however not necessary, induction of p53-dependent extrinsic apoptosis causes disruption of cross-talk interaction between Akt and ERK with cell death-related signals and tumor suppressors [20], together these crucial events represent a major pro-apoptotic stimulus. PON1-overexpressing H460 and H1299 cells had different regulation pattern in p53 phosphorylation, which can be traced with the expression of different wild-type p53 status (Figure 7F). H460-PON1 abolished both phospho- and total forms of p53, while it was null in H1299-PON1 cells. H1299-PON1 dramatically decreased total pro-caspase-3 and thus activated caspase-3, which cleaves p21 CDKI. Under STS, an ATP-competitive kinase inhibitor, we both found that there was an increase in the clearance of abnormal, dying cell fraction and knockdown of PON1 exacerbated this effect by increasing the fraction of apoptosis induced by STS (Figure 7G). In support, ATP levels were decreased in A549-shPON1 cells while ATP production is preserved in PON1-overexpressing cells even when stressed under STS (Figure 7H; Supplementary Figure 10A). This finding relates PON1 activity to extrinsic ligand-induced cell death since glycolytic shifts mediate the binding of TNF ligands and TRAIL to their respective death receptors [20]. Meanwhile, PON1 overexpression resulted in protection against ligand-stimulated caspase-3/−7 activation in response to TNF-α, TRAIL (not significant), and STS, however co-treatment with the combination of the two with STS did not produce any significant changes (Figure 7I, 7J; Supplementary Figure 10B). This suggests that the involvement of TNF-α and TRAIL in PON1-mediated escape from apoptosis is caspase-independent while STS works through a caspase-directed mechanism. In light of this, we also found a significant rescue of apoptotic fraction induced by STS in glucose-deprived H460-PON1 cells (Supplementary Figure 11). We next examined the effect of PON1 on CHOP expression involved in PON2/PON3-regulated ER-stressed triggered activation [21] in transient PON1-overexpressing cells. PON1 caused 1.4 fold down-regulation of CHOP mRNA, and attenuated the increase of STS-induced JNK mRNA (Supplementary Figure 12). Further, PON1-overexpressing H460-PON1 cells resulted in decreased p53 activation after treatment with either ETOP or STS with subsequent down-regulation of caspase-3 and -8, p27, and p21, while PON1 deficiency increased p53 and caspase-activation A549-shPON1 cells (Figure 7K). This supports the down-regulation of the endogenous phosphorylated p53 expression in PON1-overexpressing H460 cells, coordinating the p53 cell death pathway to defend cells from genotoxic and apoptotic damages induced by ETOP and STS, respectively.

### PON1 contributes to tumor growth *in vivo*

**Figure 8:**
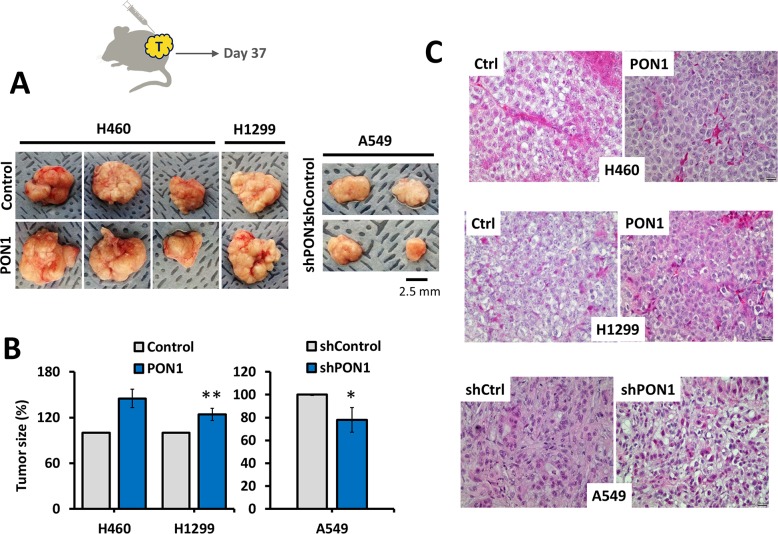
PON1 promotes tumor growth *in vivo* **(A, B)**, Schematic diagram of tumor inoculation with indicated NSCLC cells subcutaneously injected into nude mice and images taken after removing tumor xenografts grown for 37 days and measured using caliper to quantify tumor size. **(C)** Representative H&E stained tissues of indicated NSCLC-inoculated tumors. Magnifications, x200. Symbols represent mean ± S.E.M. n=4 mice per group; *P<0.05; **P>0.01. Animal experiments were biologically replicated twice.

Association of PON1 to tumor development has been limited to the occurrence of its non-synonymous gene polymorphisms in cancer patients [22, 23]. To address the role of PON1 on tumorigenesis *in vivo*, we subcutaneously injected H460-PON1, H1299-PON1, A549-shPON1 cells, and vector control cells suspended in Matrigel into the upper and lower right and left flanks of nude mice. This subcutaneous mouse xenograft experiment demonstrated that all mice (n = 4/4 per group; 16 tumors) injected with control and PON1-modified cells had multiple bilateral subcutaneous tumors within 35-37 days of inoculation, whereas no mice that were injected with PBS only showed signs of any tumor growth (n = 4; 8 sites). Tumors were assessed by caliper to measure the size and confirmed by H&E staining (Figure 8A, 8B, 8C). Condensed structure and greater tumor masses were observed in both PON1-overexpressing tumors while smaller size tumors with more spatial mass of cells were observed in PON1-knockdown A549 tumors. These results indicate that the abovementioned antioxidative, proliferative, and anti-apoptotic effects of PON1 might systematically act as mechanisms for tumor growth.

## DISCUSSION

In this study, we identified notable LC cell-autonomous function for PON1 providing evidence of regulating the response mechanism of LC cells to oxidative stress with functional consequences in LC tumor physiology, metastatic potential, and cell death. This also collectively suggests that LC might potentially exploit the antioxidative function of PON1 to benefit cancer cell growth and survival. Phenotypes observed from gain and loss of PON1 function and expression in LC cells, both at the gene and protein stability levels, also help provide a clear and direct basis for previously identified risk factor associations and polymorphisms for PON1 and their implication in response to oxidative stress across LC subtypes [24, 25]. Our unbiased analyses additionally highlighted PON1 as a potential driver of LC carcinogenesis by inducing outgrowth and invasive ability through its paracrine and cell-autonomous antioxidative function regulating adaptive response to therapy-induced apoptosis. Functional assays also suggest that this regulatory observation is an oncoprotein phenotype specific to LC cells.

In our previous efforts in studying the implication of PON1 in LC, we recently discovered that serum PON1 is a highly significant meta-marker, along with SERPINA4, for the differential diagnosis of LC and lung disease [26]. More notably, our previous integrated proteomics-based analysis of serum PON1 utilizing a wide-range of patient serum samples diagnosed with different LC sub-types indicated that the PTM glycosylation of PON1 in form of fucosylated PON1 is a highly specific biomarker for the diagnosis of SCLC [13]. How do these previous serum PON1 biomarker data fit with the tissue expression of PON1 across LC sub-types? Interestingly, we identified in this study that there is a varied PON1 protein expression pattern between our cohorts of SCC and lung adenocarcinoma patient tissue samples matched with adjacent normal tissues. Although minimal in protein overexpression, PON1 is expressed in SCC while infrequently deleted in lung adenocarcinoma. However, due to the limited number of LC patient tissues tested, we further aimed to substantiate this observation by bioinformatics analysis of *PON1* copy numbers from publicly available datasets. Mining several meta-analyses of oncogenomic/transcriptomic datasets derived from the TCGA databases, we found a higher frequency of differential gene expression (copy numbers) of *PON1* between SCC and lung adenocarcinoma cohorts across all variants per subtype. In a broad context, higher amplified copy numbers of *PON1* were detected in SCC than lung adenocarcinoma cohorts while infrequent, higher deletion frequency in lung adenocarcinoma than SCC cohorts. One can consider the possibility that depending on LC subtype, cells might inhibit the secretion of PON1 protein from the liver cells to the blood, as in our previous study [13], we found an overall reduction of serum PON1 levels in both mouse models and SCLC patients despite dramatic fucosylation levels of the protein, which in this form can persist longer in the blood due to reduced proteolytic degradation. Nonetheless, future studies will be aimed at identifying the biological relevance of this varied PON1 expression across LC subtypes using patient-derived animal models and cells to better define the implication of dramatic PON1 expression changes and its PTMs and their implication on the pathobiology of LC.

In other malignancies, PON1 being part of an endogenous free radical scavenging system has been observed to be a regulator of its expression and risk factor value in various human cancers of the endometrium, ovary, stomach, and pancreas [27, 28, 29, 30]. In addition, decreased homozygote Q allelic *PON1* activity has been observed in breast cancers while M and Q alleles of *PON1* have been regarded as high susceptibility risk factors [31, 32]. However, explicit reporting of the cancer-causing mechanism of PON1 in these cancers has not yet been elucidated despite these associations.

Molecularly, PON1 is released by a high-affinity desorption mechanism and malignancies take advantage of its regulating effects on systemic oxidative-stress to affect tumor development with specific actions in the elimination of lipid-soluble radicals from lipid peroxidation [33, 34]. Most probably, this is presumed to be accomplished by a consequence of its antioxidative function. It also seems plausible to relate PON1 function with PON2 and PON3 since recent studies have demonstrated their localization in various cancer cells and tissues and found to regulate mitochondrial cell death. Located adjacent to one another in a cluster on the long arm of human chromosome 7 and mouse chromosome 6, it is known that the three PONs have a high similarity. Among other PONs, PON1 is most known to have a dual-functioning system, hydrolytic and antioxidative functions, which can be pointed out why PON1 is a risk factor in various diseases. From this, it is worth examining if cancer cells have ways to scavenge serum PON1 and take advantage of its known metabolic functions such as antioxidative and anti-inflammatory effects. Such postulation can be generally supported by studies currently linking PON1 genotypes to lung, endometrial, ovarian, gastric, and pancreatic cancers [35, 36].

Factors have been described that are able to modulate PON1 gene expression such as some interleukins and tumor necrosis factor-α, explaining the anti-inflammatory effects induced by the enzyme in some disease contexts including cancer. Intriguing evidence has related smoking activity with decreased serum paraoxonase activity causing a presumption on low-level expression of PON1 in the serum of SCLC patients. Such events prove that simple knowledge about the *PON1* genetic variants cannot possibly be sufficient to reveal the relationship of the previously described PON1 variants to cancer risk [37].

Lines of evidence indicate that PON1 uptake in macrophages is mediated by specific binding sites with HDL which is able to suppress this event and can further be regulated by apolipoprotein A-1 [38]. However, differential processing of fucosylated and non-fucosylated PON1 uptake in any cell type has never been described before. It still needs to be tested which form of PON1 has antioxidative and anti-inflammatory function. Meanwhile, it is reported that reduced expression of the scavenger receptor B-I (SR-BI) is correlated with decreased PON1 activity and increased oxidative stress in mice with less apoptotic-protection as a consequence [39]. It is yet to be determined whether scavenger receptors deregulate the uptake of PON1 from the circulation. Our study raises a stirring possibility on the significance of identifying critical effectors in supporting speculations such as that LC cells uptake PON1 from the blood through its HDL-associated mechanism to benefit tumor growth and metabolism via its antioxidative function. Such presuppositions still need to be tested by further experiments. Interestingly, differential effects on H460-PON1 and H1299-PON1 overexpressing cells have been seen in the regulation of p53 and cell death-related signals which further explains why functional action in drug-induced apoptosis were different. This might be due to different wt-p53 status in these NSCLC cell lines.

A future study addressing the mechanism on how PON1 is differentially synthesized in LC subtypes may employ the need to utilize animal models with high expression of transgenic PON1 and PON1-knockout animals as the same can also be suggested to the other PONs, PON2 and PON3, to complete the role of PONs in cancer development although these future strategies may undergo limitations and demand cautious confirmation and further related evidence because of the high probability of PON1 as well as other PONs expression being differentially regulated in the tissues of healthy humans and cancer patients.

## MATERIALS AND METHODS

### Cell culture and animals

L132 and HEK293 cells (KCLB No. 10005 and KCLB No. 21573, Seoul, Korea; obtained in 2014; passage 3 to 20 after obtaining) were grown in DMEM with high glucose content (Invitrogen) supplemented with 10% fetal bovine serum (Thermo Scientific) and penicillin (100 units/mL)-streptomycin (100 μg/mL; Invitrogen) in a humidified incubator at 37°C and 8% CO_2_. A549, H358, H460, and H1299 cells (ATCC CCL-185, CRL-5807, HTB-177, and CRL-5803 respectively; obtained in 2012; passage 2 to 15 after obtaining except for cell senescence experiment) and Lewis lung cancer (LLC) cells (ATCC CLR-1642; obtained in 2011; passage 4 to 12) were all grown in RPMI (Invitrogen) with the same supplementation and cell culture procedure as mentioned above. Cells stably or transiently expressing PON1-GFP, stable shPON1 knockdown cells, control cells, and all wild type cells were cultured under normoxia. Animal studies were performed according to the protocols approved by the institutional animal care and use committee at the College of Veterinary Medicine, Seoul National University.

### Clinical human samples

A total of 39 pairs of tissue samples from LC patients with lung adenocarcinoma and SCC were obtained during surgery through resection of primary tumors. In addition, we employed 164 pairs of matched tissue and serum samples from LC patients with further disease-type classification (as limited and extensive disease stages of SCLC, adenocarcinoma, squamous and large cell carcinomas) described in our previous study [13]. After securing written informed consent, all patient samples were obtained and used in accordance with the institutional ethical guidelines of Seoul National University Bundang Hospital (IRB No. B-1201/143-003). All clinical samples were selected and categorized randomly from a large sample bank and the mentioned affiliate institution approved all protocols/methods adapted in this study utilizing the clinical samples. Preoperative chemotherapy was not conducted on all patients who participated. All tissue samples were classified as having LC (lung SCC or lung adenocarcinoma) or adjacent normal tissue.

### Bioinformatics

Initial query of *PON1* mRNA expression in human LC datasets was performed using Lung Cancer Explorer (UTSW QBRC/CCBSR; http://lce.biohpc.swmed.edu/lungcancer/). Oncoprints detailing genetic alterations and their association with *PON1* mRNA expression in represented TCGA datasets were obtained from cBio Portal. LC-specific *PON1* copy number analysis utilizing TCGA cohorts was done using Oncomine (see Results section). Genomic data and profile graphs were obtained using Affymetrix gene probe-set ID number for *PON1* on the given array platform. Confirmation of queried *PON1* copy number alterations was analyzed by mapping *PON1* copy numbers using TumorScape platform encompassing TCGA databases (Broad Institute; www.broadinstitute.org/tcga). The Integrative Genomic Viewer (IGV, Broad Institute) was used to determine the minimal common region and to subsequently sort the samples by both amplification and deletion of *PON1*, and then defined smallest amplification or deletion that encompassed the *PON1* locus. The correlation of *PON1* gene expression levels to patient survival lung adenocarcinoma patients was done using the KMplotter (http://kmplot.com). Patients were split by median PON1 expression and analysis was performed using the lung adenocarcinoma dataset irrespective of grade, stage, and treatment regimen. Both the overall survival and progression-free survival were analyzed.

### PON1 stable overexpression and knockdown studies

For establishing the stable knockdown of PON1, A549 cells were transfected with mouse pLKO.1-puro or pLKO.1 lentiviral based vector encoding shRNA constructs derived from the Broad Institute TRC1 library: pLKO.1-puro-shPON1 (clone IDs TRCN0000050360, TRCN0000050358). pLKO.1-Puro-shControl was used as described previously [40]. All retroviral vectors were purchased from Origene, (Rockville, USA). The respective oligo constructs were cloned in *Age*I, *Eco*RI sites of pLKO.1 with hairpin. DNA oligos were obtained from Open Biosystems (Thermo Scientific). Each shRNA-encoding vector was transfected in triplicate wells. One well was kept untreated, one with 4.5 μg/mL puromycin, and one with 0.01% DMSO. Surviving fraction of cells that were pre-exposed to puromycin were maintained in 0.1 μg/mL puromycin containing growth media. Cells were harvested on ice in 1x PBS and the samples were prepared for RT-qPCR analysis. Relative changes of *PON1* mRNA expression compared to the unstimulated pLKO.1 control were normalized to the expression of hGAPDH and quantified using the 2^−ΔΔCt^ method. For establishing the stable PON1 overexpressing cells, H460 and H1299 cells were transfected with bicistronic pLVX-EF1α-IRES-puro lentiviral expression vector (Clontech, Mountain View, CA) to simultaneously coexpress the human PON1 single mRNA transcript. The same process as described above was used for the stable transduced maintenance of mass-populations of puromycin-resistant cells highly expressing PON1. An unstimulated empty vector control was used as experimental control. The human PON1 protein level in stable PON1 knockdown and overexpressing cells were later confirmed by Western blotting.

### Western blotting, immunocytochemistry, fluorescence imaging

Preparation of human patients’ sera and tissue, cell, and tumor lysates and details of immunoblotting were described previously by our group [13]. Isolation of nuclei and cytosol was carried out using NE-PER Nuclear and Cytoplasmic Extraction Reagents (Pierce) following manufacturer's instructions. Immunocytochemistry was carried out using Zeiss LSM 780 ApoTome microscope (Carl Zeiss) to localize and observe PON1 distribution in cells. Immunofluorescence of cells was carried out to monitor ROS using dihydrorhodamine (DHR) or dichloro-dihydro-fluorescein diacetate (DCFH-DA), determine intensity of mitochondrial fragmentation using MitoSOX, and observe nuclear morphology using DAPI. All *in vitro* fluorescence studies were observed under charge-coupling device camera as described previously [41, 42, 43].

### RNA extraction and quantitative real-time PCR

Total RNa was extracted using Trizol (Invitrogen) according to the manufacturer's protocol. RNA was treated with DNA I (New England Biolabs, MA). cDNA amplification was performed using the High Capacity Reverse Transcription Kit (Applied Biosystems). Real-time (RT)-PCR, and quantitative reverse transcriptase RT-PCR were performed as described previously [43] to evaluate mRNA levels of genes of interest. qPCR primer sequences were designed using Primer 3 MIT software and sequences were chosen that spanned exon junctions. All sequences used are listed in Supplementary Table 1.

### Cell growth and death studies and cell cycle analysis

Cell growth was assessed by BrdU staining and 3-(4,5-dimethylthiazol-2-yl)-2,5-diphenyl-2H-tetrazolium bromide (MTT) colometric assay as described previously [41]. Assessment of drug- and ligand- induced cell death was carried out by quantification of the sub-G_1_ fraction of cells, annexin-V-FITC/PI or 7-AAD staining analyzed by FACS, and TUNEL assay, all as described previously [41, 42, 44]. Cell cycle progression analysis was done by fluorescence-cell activation sorting using FACSCalibur flow cytometer (BD Biosciences).

### Analysis of intracellular ROS, cytochrome *c* release, and mitochondrial membrane potential (Δψm) by flow cytometry and NAD+/NADPH assay

For the assessment of intracellular ROS, cells grown in 60 mm dishes were trypsinized, resuspended in growth media, pelleted (300 × g, 20°C, 5 min), and stained with 20 to 40 μM DCFH-DA (Sigma) for 40 min at 37°C, re-centrifuged, washed with cold PBS twice and fixed with 2% paraformaldehyde. Fixed cells were subjected to flow cytometry analysis using FACSCalibur (BD Biosciences). For the detection of mitochondrial cytochrome *c* release, a previously described method was adapted [21]. Briefly, cells were grown in 12-well dishes, trypsinized, pelleted (350 × g, 4°C, 5 min) in growth media, washed with cold PBS and re-centrifuged followed by immediate washing with cold wash buffer (PBS, 1% FCS, 0.1%. Na-Azide; all from Sigma) and fixed in cold PBS containing 4% paraformaldehyde for 20 min in 4°C. Fixed cells were washed, pelleted, and permeabilized/blocked with cold PBS containing 0.2% saponin (Sigma) and MOPC-21 mouse-IgG1κ (Sigma; with 1:10 ratio) for 5 min at RT. Cells were then incubated with mouse-anti-cytochrome *c* (clone 7H8.2C12; BD Biosciences; 1:20 in PBS; 20 min; 4°C) and conjugated with secondary antibody (AlexaFluor-647 goat anti-mouse IgG2b; Molecular Probes; 1:40 in PBS; 20 min; 4°C), washed twice, and resuspend with 100 μl 4% PFA, followed by addition of 400 μl of ice-cold PBS and immediate FACS analysis. Changes in the mitochondrial potential were assessed using the fluorescent potentiometric dye TMRE, which works by entering the cell in the form of an ester which is subsequently hydrolyzed and converted to tetramethylrhodamine, which is reversibly accumulated in the negatively charged mitochondrial matrix depending on mitochondrial transmembrane potential exhibiting potential-dependent accumulation in mitochondria. Δψm was analyzed using a previously described method [44]. Cells were washed with cold PBS and 100 nM TMRE dye was loaded for 10 to 20 min at 37°C. Cells were trypsinized, centrifuged, and harvested with cold PBS and were subjected to FACS analysis. Homogenous concentration of NADP+/NADPH based on glucose-dehydrogenase cycling reaction and ratios in cells was determined by following the manufacturer's protocol instructions (Abnova).

### Glucose modification in cells

Adherent cells were maintained in RPMI 1640 in high-glucose medium and medium was replaced after 48 h with glucose-deprivation medium, which was depleted of glucose (0 mM glucose, Life Technologies, Grand Island, NY) after washing with glucose-deprived medium. Glucose supplementation and deprivation in cells were adapted in same procedure in details as published previously [42].

### Tumor xenograft assays and immunohistochemistry

Bulk cell suspensions were injected subcutaneously with a 25 gauge needle into the upper and lower right and left flanks of male BALB/c nude mice (n=4; per tumor cell line type). The tumor growth curves were determined by measuring the tumor size. All mice were mercifully killed at day 35 and the tumors were removed and scaled. Tumorigenicity experiments were performed as described before [13, 45]. Tumors tissues were washed with PBS and fixed with 4% paraformaldehyde for immunohistochemistry (IHC) studies. For IHC, 4 μM thick sections were cut from formalin-fixed paraffin-embedded tissues. Staining was carried out using H&E staining according to the protocol provided by the manufacturer (Roche Diagnostics).

### Software and statistical analysis

Densitometry data from expression studies were statistically analyzed using non-parametric Kruskal-Wallis test. β-actin variations apparent in some blots were considered when calculating for densitometric values used for sample normalization. All analyses were performed using OriginPro 8.0 (OriginLab Corp.) and *p* values ≤ 0.05 were considered statistically significant. All results were obtained from at least three independent experiments and most were biologically replicated. The data are presented as mean ± SD. Statistical evaluation was done using one-/two-way ANOVA with Bonferroni's multiple comparisons post-test and Student's t test and x^2^ test. GraphPad Prism 5.01 software was used to calculate the MFI of all fluorescence images. For image analysis, the extent of apoptosis quantification by TUNEL assay and DAPI fluorescence studies were quantified with the aid of open-source image processing program ImageJ and software plugins (NIH). To quantify nuclei condensation and size, the probability density function was calculated by analyzing the pixel per pixel value of the acquired image of DAPI-stained nuclei and results were displayed as Maple distribution using Maplesoft (Waterloo Maple Inc., ON, Canada). Other methods not listed here are detailed in Supplementary Methods.

## SUPPLEMENTARY MATERIALS FIGURES AND TABLES


