# Supplementary material for: Paraoxonase-1 (PON1) induces metastatic potential and apoptosis escape via its antioxidative function in lung cancer cells
Source: Oncotarget. 2017 Apr 12;8(26):42817–35. doi: 10.18632/oncotarget.17069 (PMC5522108; doi:10.18632/oncotarget.17069)
Supplement: Supplementary file 1 [file oncotarget-08-42817-s001.pdf]

# Paraoxonase-1 (PON1) induces metastatic potential and apoptosis escape via its antioxidative function in lung cancer cells

## SUPPLEMENTARY METHODS

### Immunocytochemistry, fluorescence imaging, and nuclei size assessment

PON1 subcellular localization and various dye immunofluorescences in several cell lines were observed using Zeiss LSM 780 ApoTome microscope (Carl Zeiss, Jena, Germany), using a Plan/Apochromat x 63/1.4 oil DIC objective. For the labeling of PON1 protein, fluorescein isothiocyanate (FITC; Sigma-Aldrich) was dissolved in dimethyl formamide (Merck, Kenilworth, NJ) and was added drop wise to the PON1 cell suspension and then incubated for 1 h at 37°C, separated from secondary antibody conjugation. Cells stably expressing PON1, cells with silenced PON1 transcription, control cells, and wild type cells were seeded in Lab-Tek chamber slides (Nunc) overnight, washed with HBSS/PBS, and all incubated at room temperature unless otherwise specified. The cells were then fixed with 4% paraformaldehyde (in PBS) for 15 min and then blocked with 1% BSA (in PBS) for 1 h at room temperature followed by the incubation with human anti-PON1 primary antibody at 4°C overnight. The cells were then washed for additional two times. Permeabilization with Triton X-100 was cross-examined since PON1 can also be found in membrane-bound channels of cells. Following overnight incubation, cells were incubated with FITC-conjugated secondary anti- rabbit antibody for 2-3 h at room temperature. DAPI (0.5 µg/ml) was used to counterstain the nuclei and stored at 4°C before microscopy. Appropriate wavelengths were sequentially scanned; absence of cross-emission was also verified. For dye immunofluorescence studies, cells were cultured on black optical-bottom 96-well plates. To monitor ROS, cells were incubated in culture medium with 5 µM dihydrorhodamine (DHR) 123 or 10 µM dichloro-dihydro-fluorescein diacetate (DCFH-DA) for 30 min at 37°C, or 10 µM MitoSOX for 30 min, to determine mitochondrial fragmentation, followed by 1 h wash out at 37°C. The emission and excitation wavelengths were: 500/535 nm (DHR 123); 548/588 nm (MitoSOX). To quantify changes in nuclear size or occurrence of condensation, nuclei were stained with DAPI and images were captured using a charge-coupling device camera and the fluorescence was examined as described previously [4].

### Preparation of nuclear and cytosolic cell fractions and PON1 localization

Cells were harvested at 80% confluence through trypsination. Isolation of nuclei and cytosol was carried out using NE-PER Nuclear and Cytoplasmic Extraction Reagents (Pierce, Bonn, Germany) following manufacturer's instructions. All steps were performed on ice or at 4°C, unless stated otherwise. Protease inhibitors and reducing agents (1 mM dithiothreitol and 1 mM phenylmethylsulphonyl uoride) were added to each buffer prior to use. Briefly, hypotonic Buffer A was first prepared (20 mM HEPES with pH 7.9, 1.5 mM $MgCl_2$, 10 mM KCl) where the cells were later on incubated in 10 volumes on ice for 15 min and homogenized. Nuclei were recovered as pellet after centrifugation at 16000 x *g* for 5 min, and the supernatant was collected as the cytosolic extracts. The nuclei were extracted using Buffer C (20 mM HEPES with pH 7.9, 25% glycerol, 420 mM NaCl, 0.2 mM EDTA, 1.5 mM $MgCl_2$) for 40 min on ice. Centrifugation at 16000 x g was further conducted to remove insoluble materials, and the supernatant was used as the nuclear extract. Probes were solved in Laemmli sample buffer at a final concentration of 1 x $10^6$/mL and stored at -20°C before SDS-PAGE. Nuclear and cytosolic protein amounts were quantified by Bradford assay and were subjected to Western blotting for the localization of PON1 using anti-human PON1 antibody (used at 1:1000).

### Transient PON1 transfection

Cells were dual transfected with pCMV-GFP and pCMV-GFP-PON1 using Lipofectamine 3000 (Life Technologies). The wild type PON1 expression vector consists of full-length PON1 cDNA within the pCMV-GFP mammalian expression vector (Stratagene, La Jolla, CA). The method used for transient gene transfection was described previously [5].

### Expression detection of apoptosis, cell survival, and redox-related signals by Western blotting

Preparation of lysates, SDS-PAGE, and Western blotting were performed as described above. Rabbit anti-p21 (waf1/cip1) was used at 1:800, rabbit anti-p16INK4a was used at 1:2000, rabbit anti-phopho-Akt (ser473) and anti-Akt were used as 1:1000, mouse anti-phospho-p53 (ser15) and rabbit anti-p53 were used at 1:1000, rabbit anti-cleaved-caspase-3 and anti-cleaved-8 were used at 1:800 (all were from Cell Signaling Technology). Rabbit anti-p27, anti-phospho-ERK1/2, anti-ERK1/2 were used at 1:1000 (Santa Cruz). HRP-conjugated secondary antibodies were all used at 1:2000 (Sigma).

Other methods not listed here such as cell migration and invasion assays, scratch-wound healing assay,

determination of ATP content, caspase activity, and mitochondrial superoxide production assessment [6-8].

## REFERENCES


1. Weiss J, Sos ML, Seidel D, Peifer M, Zander T, Heuckmann JM, Ullrich RT, Menon R, Maier S, Soltermann A, Moch H, Wagener P, Fischer F, et al. Frequent and focal FGFR1 amplification associates with therapeutically tractable FGFR1 dependency in squamous cell lung cancer. Sci Transl Med. 2010; 2: 62ra93.
2. Garber ME, Troyanska OG, Schluens K, Petersen S, Thaesler Z, Pacyna-Gengelbach M, van de Rijn M, Rosen GD, Perou CM, Whyte RI, Altman RB, Brown PO, Botstein D, et al. Diversity of gene expression in adenocarcinoma of the lung. Proc Natl Acad Sci USA. 2001; 98: 13784-13789.
3. Hou J, Aerts J, den Hamer B, van Jicken W, den Bakker M, Riegman P, van der Leest C, van der Spek P, Foekens JA, Hoogsteden HC, Grosveld F, Philipsen S. Gene expression-based classification of non-small cell lung carcinomas and survival prediction. PLoS ONE. 2010; 5: e10312.
4. Aldonza MB, Hong JY, Bae SY, Song J, Kim WK, Oh J, Shin Y, Lee SH, Lee SK. Suppression of MAPK signaling and reversal of mTOR-dependent MDR1-associated multidrug resistance by 21α-methylmelianodiol in lung cancer cells. PLoS ONE. 2015; 10: e0127841.
5. Leviev I, Deakin S, James RW. Decreased stability of the M54 isoform of paraoxonase as a contributory factor to variations in human serum paraoxonase concentrations. J Lipid Res. 2001; 42: 528-535.
6. Na SS, Aldonza MB, Sung HJ, Kim YI, Son YS, Cho S, Cho JY. Stanniocalcin-2 (STC2): a potential lung cancer biomarker promotes lung cancer metastasis and progression. Biochim Biophys Acta. 2015; 1854: 668-676.
7. Witte I, Altenhöfer S, Wilgenbus P, Amort J, Clement AM, Pautz A, Li H, Förstermann U, Horke S. Beyond reduction of atherosclerosis: PON2 provides apoptosis resistance and stabilizes tumor cells. Cell Death Dis. 2011; 2: e112.
8. Ahn JM, Sung HJ, Yoon YH, Kim BG, Yang WS, Lee C, Park HM, Kim BJ, Kim BG, Lee SY, An HJ, Cho JY. Integrated glycoproteomics demonstrates fucosylated serum paraoxonase 1 alterations in small cell lung cancer. Mol Cell Proteomics. 2014; 13: 30-48.

# SUPPLEMENTARY FIGURES AND TABLE

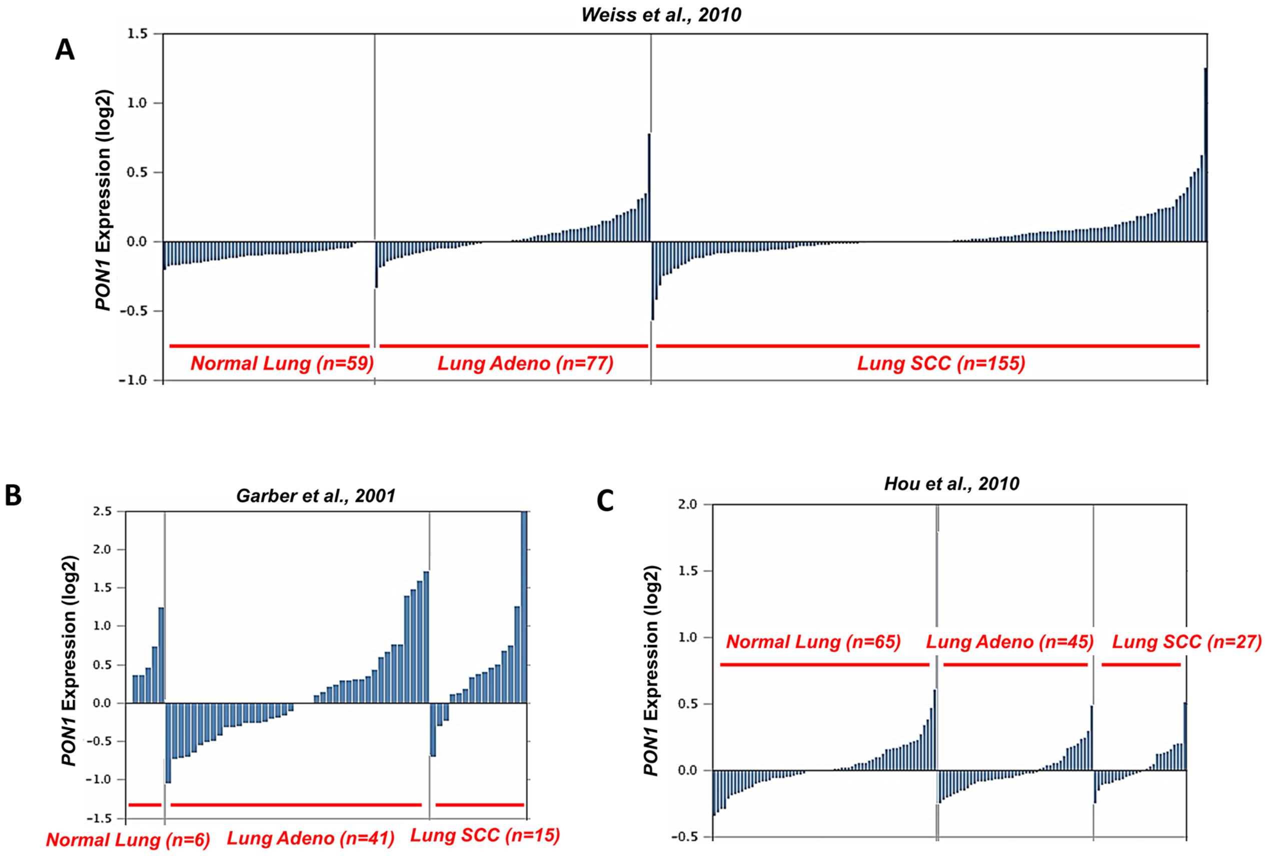


**Supplementary Figure 1: Frequent variability of PON1 gene expression between human lung squamous cell carcinoma and lung adenocarcinoma tumors, related to Figure 1.** Expression levels of *PON1* mRNA across independent published datasets [1, 2, 3]. Individual log2 transformed values are expressed showing high degree of variability of *PON1* mRNA across LC subtypes: lung SCC and lung adenocarcinoma and their specific variants. Expressions were analyzed using Oncomine (http://www.oncomine.org).

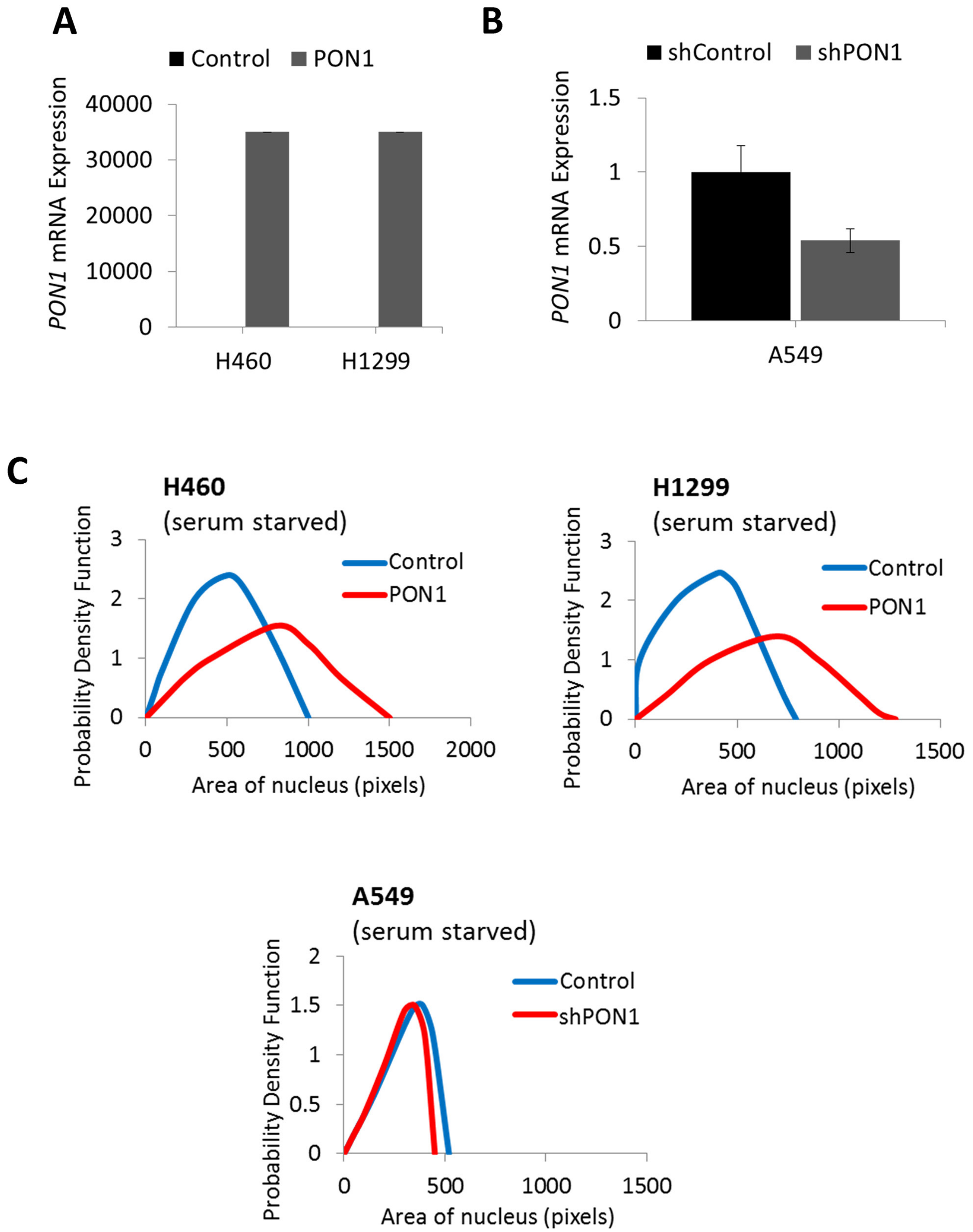


**Supplementary Figure 2: PON1 expression can be genetically engineered in lung cancer cell culture systems, related to Figure 2. (A, B)** Stable transfection efficiency test of PON1 gene overexpression in H460 and H1299 cells and PON1 gene knockdown in A549 cells through real-time qRT-PCR. Symbols represent mean ± S.E.M. **(C)** Nuclei area size of day 5 serum-starved cells (same condition as in Figure 3A) was determined by analyzing pixel per pixel size of the pseudocolored blue fluorescence of DAPI staining (n=8 images).

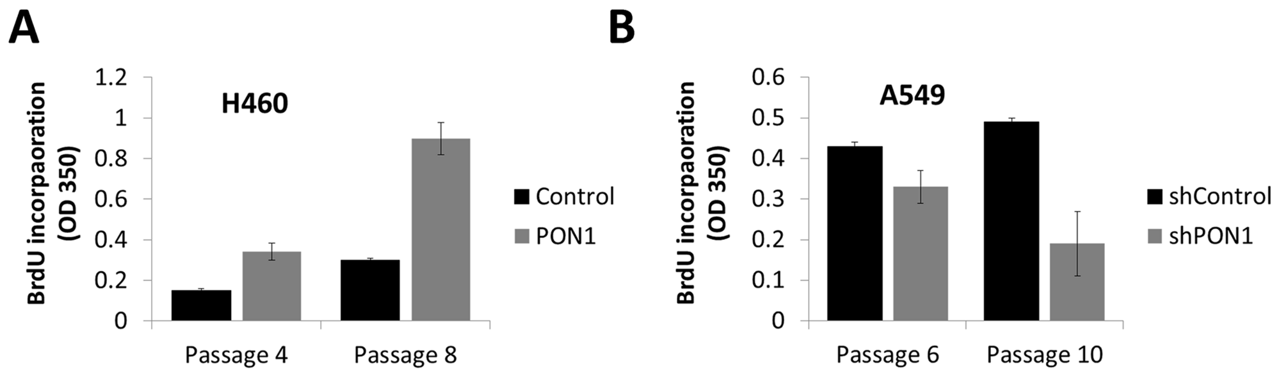


**Supplementary Figure 3: PON1 promotes increased DNA synthesis in lung cancer cells, related to Figure 3.** (**A, B**) Colometric BrdU incorporation of H460-PON1, H1299-PON1, A549-shPON1, and their respective control cells. BrdU was incorporated into the DNA of proliferating cells and cells were further incubated with an anti-BrdU antibody conjugated to peroxidase. Bound BrdU is detected by a substrate reaction and quantified by absorbance measurement at 350 mm. Symbols represent mean ± S.E.M.

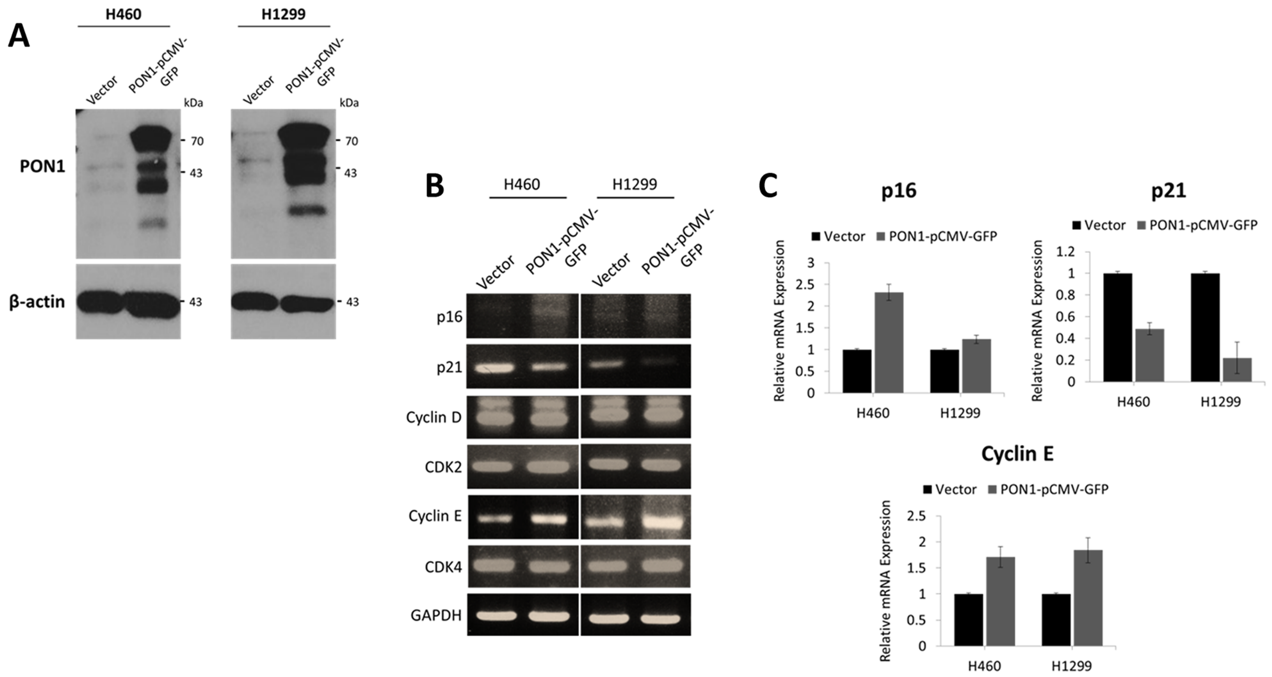


**Supplementary Figure 4: Transient transfection of PON1 and effect on the expressions of cell-cycle-related genes, related to Figure 3.** (**A**) Protein blotting of vector control and transiently PON1-transfected H460 and H1299 cells (PON1-pCMV-GFP) showing transfection overexpression efficiency. (**B**), The pCMV-GFP plasmid was utilized to deliver the PON1 gene appearing as double-band with size in between 39 to 70 kDa. mRNA expressions of p16, p21, cyclin D, CDK2, cyclin E, and CDK4 in vector and PON1-pCMV-GFP transfected H460and H1299 cells determined by semi-quantitative RT-PCR. (**C**), mRNA gene levels of p16, p21, and cyclin E were determined by real-time RT-PCR. All gene levels were normalized to GAPDH. For one experiment, three assays were carried out and only one set of gels is shown. Symbols represent mean ± S.E.M.

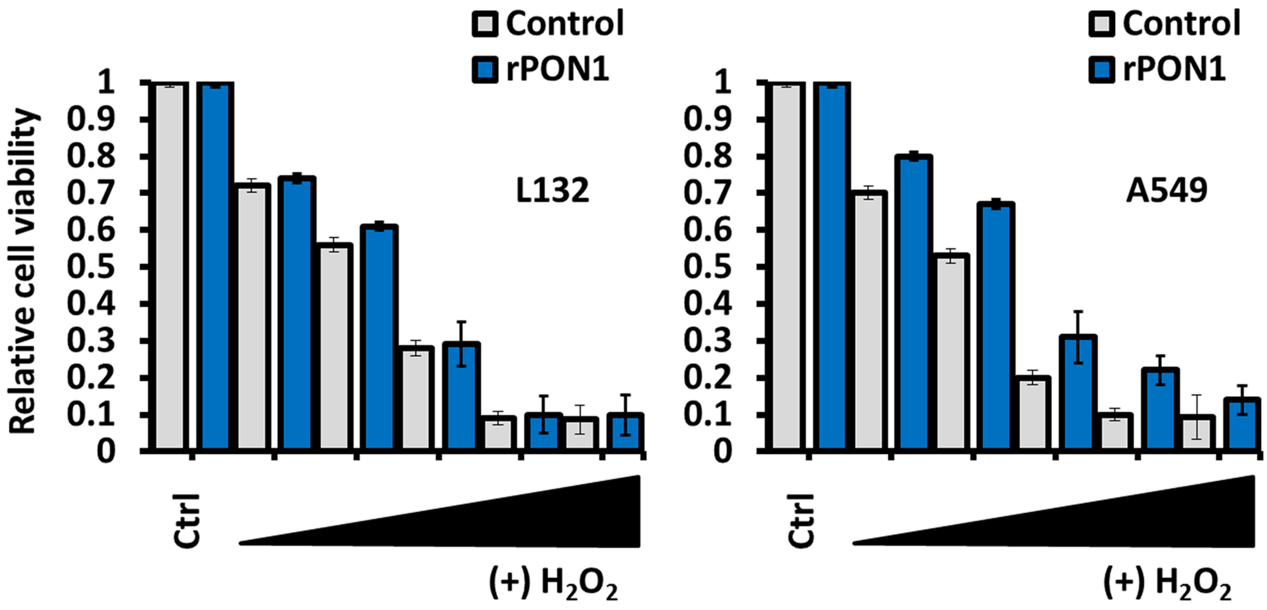


**Supplementary Figure 5: Recombinant PON1 treatment defends lung cells from H2O2-induced oxidative stress, related to Figure 4.** L132 and A549 cells were treated with different concentrations of $H_2O_2$ for 24 h with or without 50 ng/ml rPON1 in medium. Cell viability was assessed by MTT assay.

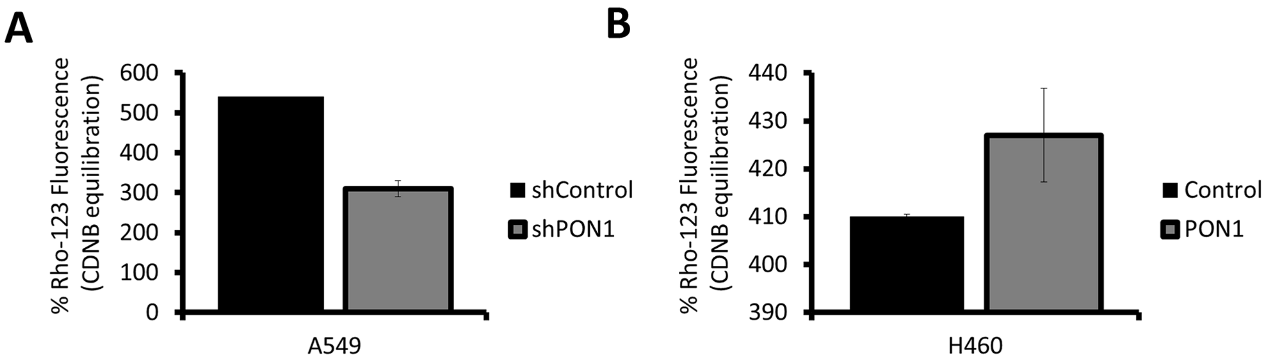


**Supplementary Figure 6: PON1 supports increase of intracellular ROS in lung cancer cells, related to Figure 4.** **(A, B)** Summary bar graphs of fluorescence signal from Rho-123 in transfected A549-shPON1, H460-PON1, and their respective control cells (300-400 cells pooled from three independent experiments) analyzed by flow cytometry after equilibration with 0.5 mM CDNB. Symbols represent mean ± S.E.M.

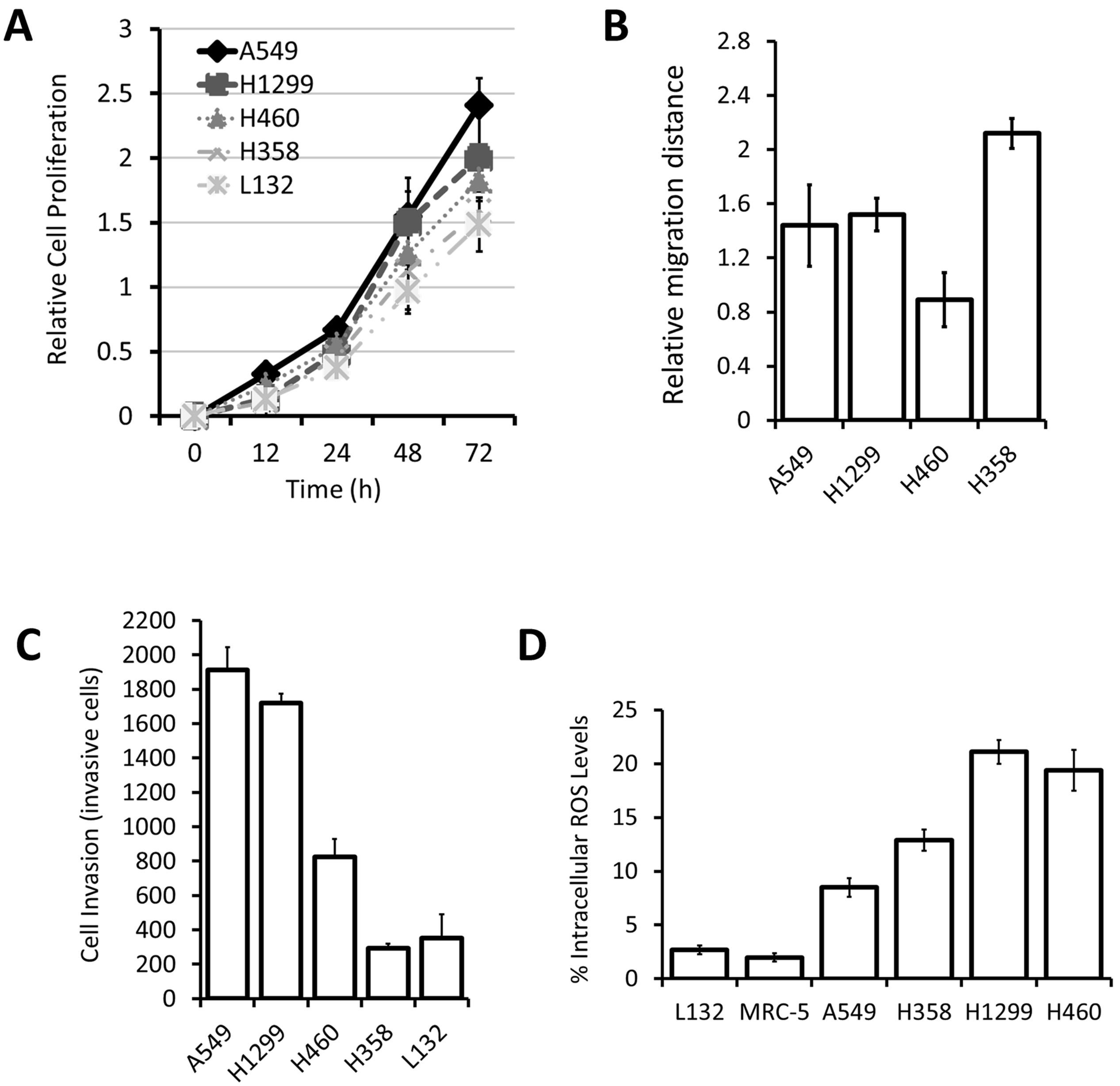


**Supplementary Figure 7: Lung normal and cancer cell lines display differential ROS levels and metastatic activity, related to Figure 5. (A)** Cell proliferation rate of respective wild-type lung normal and cancer cells analyzed through colometric MTT assay. **(B)** Cell migratory activity of lung normal and cancer cells analyzed through scratch-wound healing assay. Migration was assessed after wounding the confluent cell plate and observed after 24 h. **(C)** Cell invasion activity of lung normal and cancer cells assayed in a chamber with matrigel as base membrane. Invasive cells were assessed in the second base of the chamber and stained with crystal violet for counting. **(D)** Relative intracellular ROS levels of lung normal and cancer cells analyzed through flow cytometry after exposure to DCFH-DA dye. Symbols represent mean ± S.E.M.

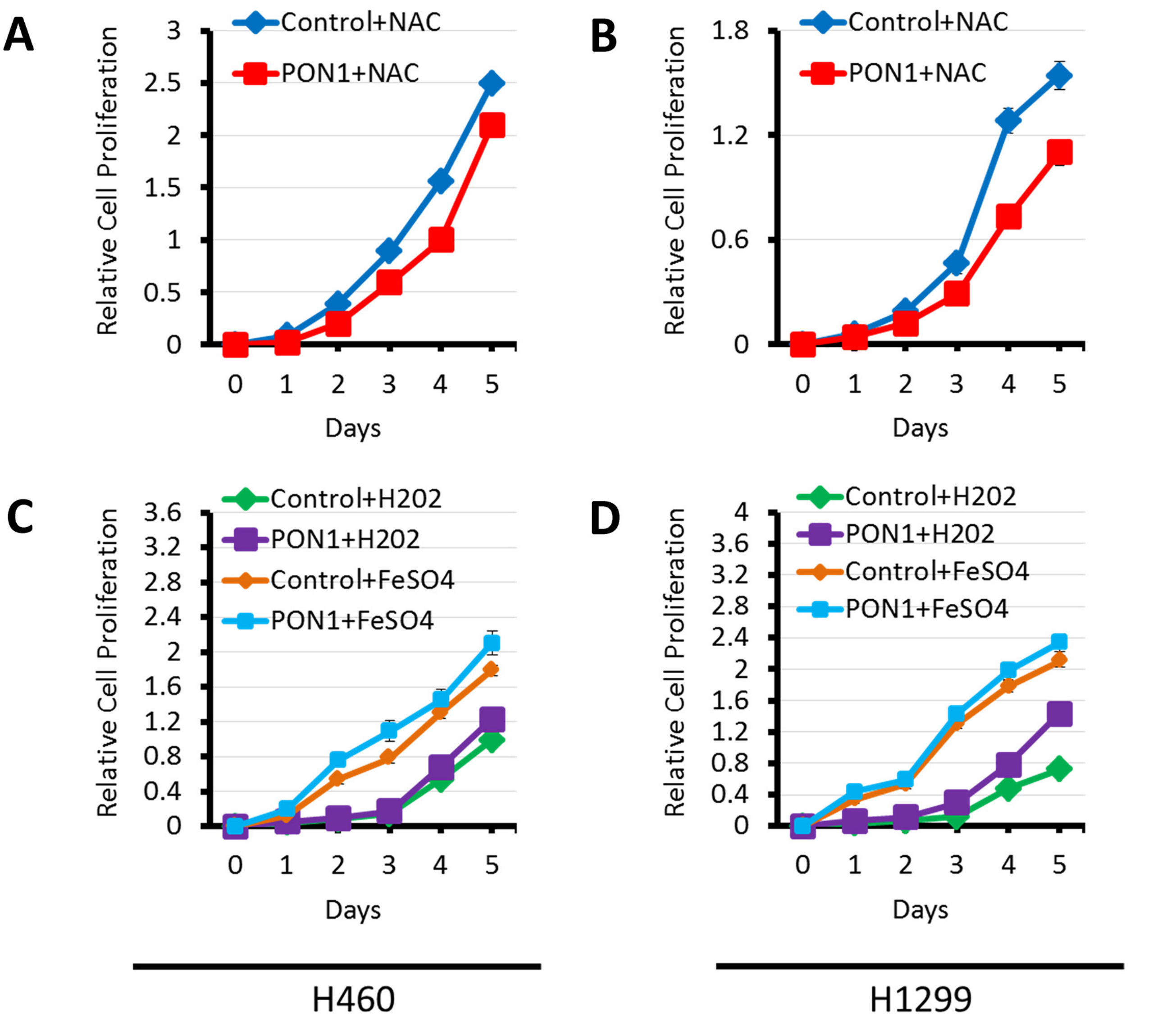


**Supplementary Figure 8: PON1 regulates lung cancer cell growth through ROS mechanism, related to Figure 5. (A, B, C, D)** Cell growth activities of H460-PON1, H1299-PON1, and control cells assessed by MTT assay after binary time-dependent treatments with either ROS inhibitor NAC or ROS stimulators $H_2O_2$ and $FeSO_4$. Symbols represent mean ± S.E.M.

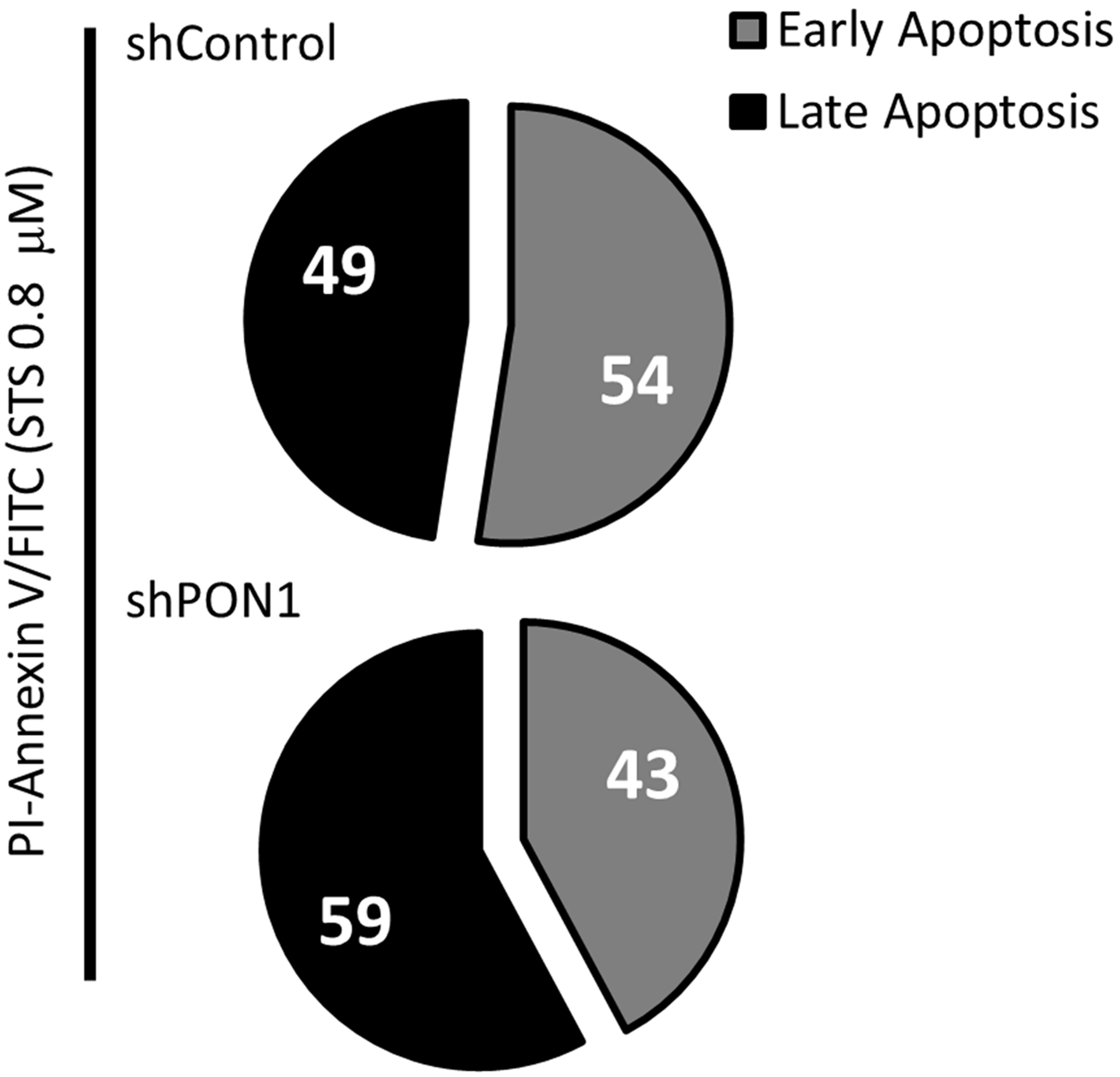


**Supplementary Figure 9: Knockdown of PON1 increases late apoptotic fraction of lung cancer cells in response to drugs, related to Figure 7.** FACS-based identification of early and late apoptosis from fluorescent activated cells after double-staining by annexin-V-FITC/PI for 24 h. A549-shPON1 and A549-shControl cells were treated with either 0.8 µM STS for 24 h before subjecting to FACS.

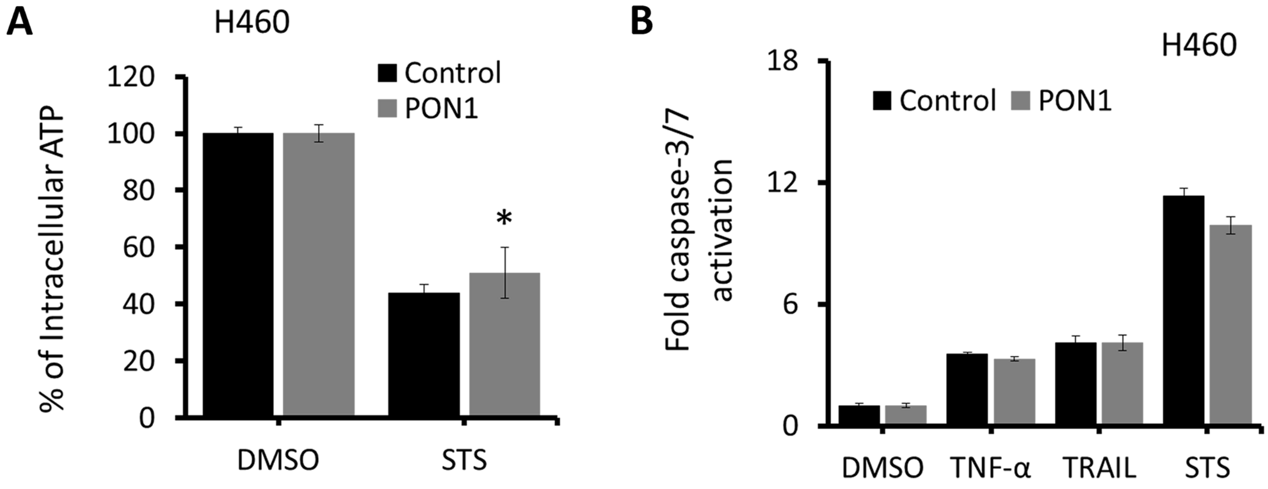


**Supplementary Figure 10: PON1 display anti-apoptotic effects in lung cancer cells, relative to Figure 7.** **(A)** Intracellular ATP level determination in H460-CTL and H460-PON1 cells after treatment with either DMSO or STS for 16 h in culture. **(B)** Caspase 3/7 activity of H460-CTL and H460-PON1 cells after treatment with DMSO, TNF-α, TRAIL, or STS for 16 h in culture. Symbols represent mean ± S.E.M. **P*<0.05.

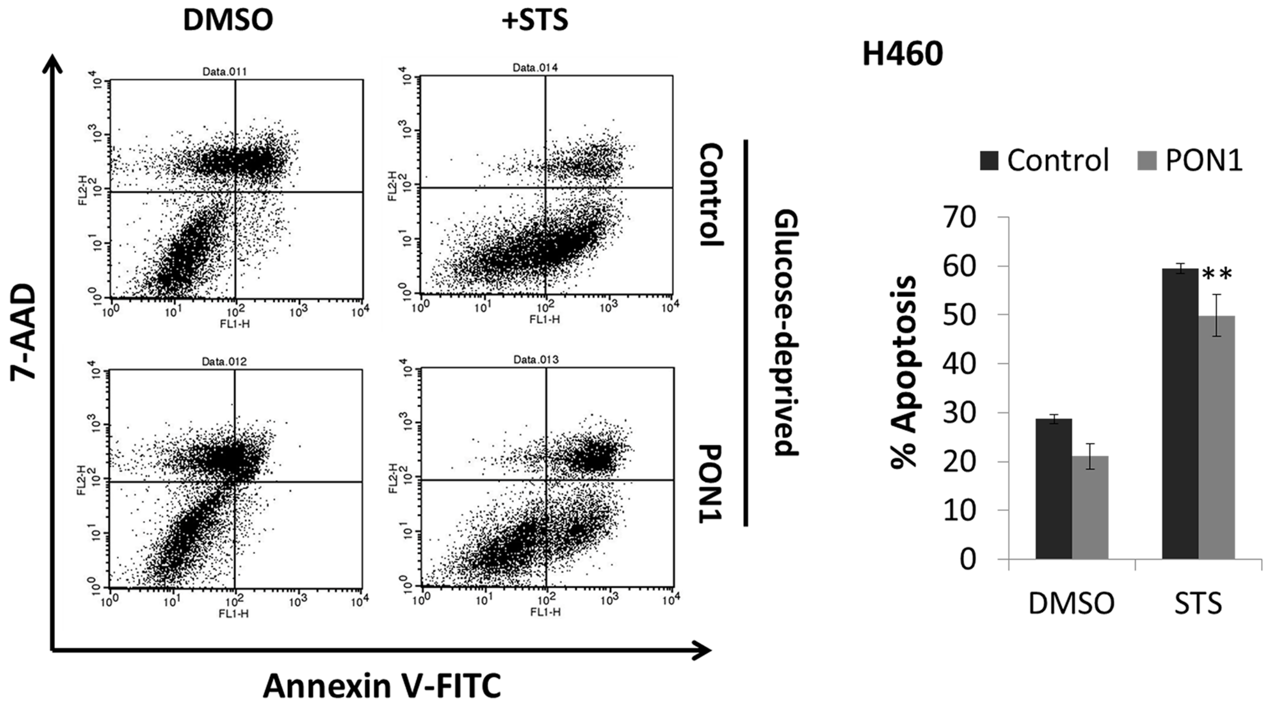


**Supplementary Figure 11: PON1 regulates glucose-deprivation-induced ligand-cell-death in lung cancer cells.** H460-PON1 and control cells were glucose-deprived for 12 h and were subsequently treated with DMSO or 0.8 μM STS for 24 h. Cells were subjected to FACS analysis to determine apoptosis by double-staining using annexin-V-FITC/7-AAD.

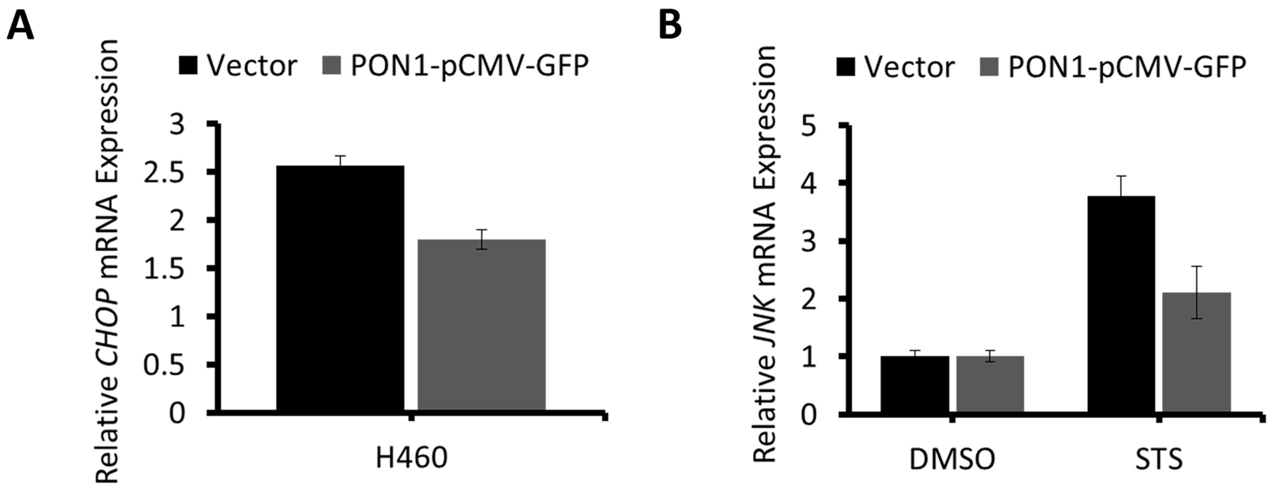


**Supplementary Figure 12: PON1 attenuates STS-induced JNK downregulation in lung cancer cells.** **(A)** CHOP gene mRNA levels in transiently expressing vector and H460-PON1 assessed through semi-quantitative real-time RT-PCR. **(B)** JNK gene mRNA levels in transiently expressing vector and PON1 H460 cells after treatment with either DMSO or STS for 16 h in cultures assessed through semi-quantitative real-time RT-PCR. Symbols represent mean ± S.E.M.

**Supplementary Table 1: Primer sequences used in real-time PCR, quantitative real-time reverse-transcriptase-PCR (qRT-PCR), and/or ELISA**

| Gene | Forward Sequence (5' to 3') | Reverse Sequence (5' to 3') |
|---|---|---|
| *PON1* | GGATCCATGGCGAAGCTGATTGCGTCTAC | GCGGCCGCGAGCTCACAGTAAAGAGCTTTG |
| *p16* | GAACTCTTTCGGTCGTACCC | CGAATCTGCACCGTAGTTGA |
| *p21* | ATGTGTCCTGGTTCCCGTTTC | CATTGTGGGAGGAGCTGTGA |
| *cyclin D* | AAGCTGTGCATCTACACCGA | CTTGAGCTTGTTCACCAGGA |
| *cyclin E* | CGGGTCCACAGGATGCGAAGGA | CAGGTGTGGGGATCAGGGAGCA |
| *CDK2* | CATTCCTCTTCCCCTCATCA | CAGGGACTCCAAAAGCTCTG |
| *CDK4* | CATGTAGACCAGGACCTAAGG | AACTGGCGCATCAGATCCTAG |
| *CHOP* | CATCACCACACCTGAAAGCA | TCAGCTGCCATCTCTGCA |
| *JNK1* | GCGCGGATCCTTGCTTGCCATCATGAGCAG | GCGCGGATCCCAGACGACGATGATGATGGA |
| *β-actin* | AGGACCGAGTCAGGAACGA | CTGTGAAACCTCGGGAATA |
| *GAPDH* | ATCCCATCACCATCTTCCAG | CCATCACGCCACAGTTTCC |
